# Bacteriocin: A natural approach for food safety and food security

**DOI:** 10.3389/fbioe.2022.1005918

**Published:** 2022-10-24

**Authors:** Dibyajit Lahiri, Moupriya Nag, Bandita Dutta, Tanmay Sarkar, Siddhartha Pati, Debarati Basu, Zulhisyam Abdul Kari, Lee Seong Wei, Slim Smaoui, Khang Wen Goh, Rina Rani Ray

**Affiliations:** ^1^ Department of Biotechnology, University of Engineering and Management, Kolkata, India; ^2^ Department of Biotechnology, Maulana Abul Kalam Azad University of Technology, Kolkata, India; ^3^ Department of Food Processing Technology, Malda Polytechnic, West Bengal State Council of Technical Education, Govt of West Bengal, Malda, India; ^4^ NatNov Bioscience Private Limited, Balasore, India; ^5^ Skills Innovation and Academic Network (SIAN) Institute, Association for Biodiversity Conservation and Research (ABC), Balasore, India; ^6^ Department of Agricultural Sciences, Faculty of Agro-Based Industry, Universiti Malaysia Kelantan, Jeli Campus, Kelantan, Malaysia; ^7^ Laboratory of Microorganisms and Biomolecules, Center of Biotechnology of Sfax, Sfax, Tunisia; ^8^ Faculty of Data Science and Information Technology, INTI International University, Nilai, Malaysia

**Keywords:** preservatives, shelf-life, food spoilage, bacteriocins, lab

## Abstract

The call to cater for the hungry is a worldwide problem in the 21st century. Food security is the utmost prime factor for the increasing demand for food. Awareness of human health when using chemical preservatives in food has increased, resulting in the use of alternative strategies for preserving food and enhancing its shelf-life. New preservatives along with novel preservation methods have been instigated, due to the intensified demand for extended shelf-life, along with prevention of food spoilage of dairy products. Bacteriocins are the group of ribosomally synthesized antimicrobial peptides; they possess a wide range of biological activities, having predominant antibacterial activity. The bacteriocins produced by the lactic acid bacteria (LAB) are considered to be of utmost importance, due to their association with the fermentation of food. In recent times among various groups of bacteriocins, leaderless and circular bacteriocins are gaining importance, due to their extensive application in industries. These groups of bacteriocins have been least studied as they possess peculiar structural and biosynthetic mechanisms. They chemically possess N-to-C terminal covalent bonds having a predominant peptide background. The stability of the bacteriocins is exhibited by the circular structure. Up till now, very few studies have been performed on the molecular mechanisms. The structural genes associated with the bacteriocins can be combined with the activity of various proteins which are association with secretion and maturation. Thus the stability of the bacteriocins can be used effectively in the preservation of food for a longer period of time. Bacteriocins are thermostable, pH-tolerant, and proteolytically active in nature, which make their usage convenient to the food industry. Several research studies are underway in the domain of biopreservation which can be implemented in food safety and food security.

## Introduction

The globalization of the food trade has stipulated for food production the distribution of food products from centralized production corporations and storage of food products on a large scale. Food safety and food security have become a worldwide concern, at the same time as an increase in the population. Fresh foods and minimally processed foods present a new challenge to food safety and security by inhibiting food-borne pathogens and other microbes. Generally, food-preservation processes usually include cold storage, drying, salting, and thermal sterilization. Canning, pasteurization, and the use of chemical additives are some modern techniques that have been used for food preservation by increasing their shelf life. According to food safety standards, strict food ratification, and consumer demands, some classical preservation techniques have been rejected, including the addition of salt and some preservatives namely acetic acid, benzoic acid, and sorbic acids to the foods. These preservation techniques showed some allergic response in consumers; also, this leads to the formation of carcinogenic products from nitrites i.e., nitrosamines (Haddad Kashani et al., 2012). These have drawn attention to the establishment of the alternative biopreservation technology. The utilization of nonpathogenic microorganisms and their metabolic products ensures food safety and enhances its shelf life through the inhibition of food-borne pathogens preventing the spoilage of food. In recent times, the rising demand for biopreservation techniques, selection, improvement, and production of beneficial microbial products have gained importance in various food industries.

In order to search for various biopreservatives as alternative to the use of chemical preservatives, bacteriocins have aroused attention, to be used as new-era food preservatives. Bacteriocins are the groups of ribosomally secreted antimicrobial peptides possessing the ability to kill or inhibit bacterial strains which are closely related or non-related, but cause no harm to themselves (Leroy and De Vuyst, 2004). Nisin is the first FDA-approved bacteriocin that has been used in the preservation of pasteurized processed cheese spread (Kierończyk et al., 2020). Lactic acid bacteria (LAB) are considered the preferred source for the bacteriocins as they create no cytotoxic effect to consumers. Apart from LAB, some *Bacillus sp.* have been reported for the synthesis of bacteriocin. Based on evolution, the synthesis of one or more than one bacteriocin is found to be extremely advantageous. The elimination of competing organisms from the environmental context is found to be optimistic for the species’ diversity and expeditious bacterial growth (Dykes, 1995; Kirtonia et al., 2021). It has been observed that low molecular weight antibiotics like tetracyclines, bacteriophage, bacteriolytic enzymes, hydrogen peroxide, toxins, lytic agents, and some metabolic by-products showed equivalent functions of bacteriocins. Although the bacteriocins contain intrinsic effects against bacterial population, their effectiveness is found to be diverse in mixed populations like biofilms.

## The gene cluster of gram-positive and gram-negative bacteria

Most of the gram-positive bacteria are responsible for bacteriocin production. For example, bacteriocin like nisinA and pediocin PA1 belong to Class I and Class II and have operonic structures. The gene cluster of nisinA contains 11 open-reading frames, analogous to lantibiotics. The structural gene nisA along with collateral functional genes including modification enzyme genes nisB and nisC, immunity gene nisI, peptidase gene nisP, and translocating enzyme gene nisT are regulated by the single promoter. The nisB and nisC genes regulate the post-translational modification *via* generating Dha and Dhb and fabricating lanthionine bridges (Koponen et al., 2002). The cleavage of the leader sequence from the pre-peptide occurred through nisP, followed by the transportation of the modified peptide across the membrane through nisT (Siezen et al., 1995). The immunity protein nisI is lipopeptide in nature and responsible for the immunity of the intracellular nisin. The downstream promoter regulates the other three immunity genes nisFEG and hinders the interaction between nisin and the producer cell membrane (Stein et al., 2003). NisR and nisK are the middle encoder genes which regulate the response for signal transduction and the histidine protein kinase.

Enzyme modification navigates the alteration among the gene clusters of lantibiotics. In lacticin A, a unique enzyme carries out the gene modification. This lacM enzyme combats the nisB and nicC analogous protein and conciliates the duplication of lanM gene in lacticin operon (McAuliffe et al., 2001).

The pediocin operon is typically present in Class II bacteriocins emerging from multiple species of *Pediococcus* and the group of *Lactobacillus plantarum*. The Class II bacteriocins operon is analogous to the colicin operon and has some diversification compared to the lantibiotics, as few enzymes are involved in post-translational modifications. PapA is the subsequent structural gene of the promoter, followed by immunity protein papB, and papC a peptidase which usually cleaves to the leader sequence and a transporter gene papD (Fimland et al., 2005).

The plasmid-encoded genes are horizontally transferred, and are specified through the diversification of pediocin-producing bacteria (Gebhard, 2012). Although the pediocin operon present in several *Pediococcus* species is homologous to the gene cluster of *L. plantarum*, there are some deviations in the operon sequence at the hundred base pairs on either side of the operon. The pediocin operon contains a cluster of four genes papABCD, with an additional satellite gene leading to genetic exchange (Kotelnikova and GelfandBacteriocin, 2002).

Although there are various bacteriocin structures and sequences present, the operon structure and sequence manifest the homology between distantly related bacterial species. It was observed that the secretion machinery of microcins is equivalent to that of the foramen colicins. Contemporary computational biological work revealed that Gram-negative AMPs have a similar expression system of Class-II (Dirix et al., 2004). The processing machinery of microcin has a parallel functional similarity to the AMP streptolysin enzymes (Lee et al., 2008). The SOS promoter regulates the colicin operons which are nuclease active. Some regulatory mechanisms including proteolytic processing are not considered under these systems. Colicin is one of the Gram-negative bacteriocins that has incongruity to the bacteriocins of lactic acid bacteria (LAB). Colicin has a heterogeneous structure present in operon which administers the immunity genes (Cascales et al., 2007). The immunity gene *cxi* is complemented to the structural gene of colicin and is able to permeabilize the cell membrane of the target. Along with structural genes, the immunity genes are co-transcribed in colicins. Similar to the Class II bacteriocins, the immunity gene is present immediately after the structural genes. The structural gene is phrased as *cxa* (“x” varies depending upon the letter code for colicin, i.e., colicin V and colicin E1) based on the cell surface receptor and is present at the downstream of the promoter. However, there are few additional processing genes present in the operon. The colicin operon encodes the lysin *via cxl* and pioneers the bacteriocin release from the producer cell (Riley and Wertz, 2002).

## Classification of bacteriocin

Bacteriocins have been categorized into different classes given the various standards like molecular sizes, physical properties, producer organisms, and mechanism of action. Nonetheless, there is no definite arrangement. In 1993 Klaenhammer categorized bacteriocins into four classes (Klaenhammer, 1993). In this grouping, class I is lantibiotics portrayed by thermostable properties, extremely low molecular weight (<5 kDa), and presence of lanthionine and its derivatives. Nisin can act as an example for all the members of this class. Few thermostable peptides without lanthionine derivatives are present in the class and have a molecular weight up to 10 kDa. It additionally incorporates three subclasses as IIa (pediocin and enterocin), IIb (lactocin G), and IIc (lactocin B). Class III accumulates high molecular weight (>30 kDa) thermolabile peptides, and class IV contains enormous peptides joined with carbohydrates or lipids (Klaenhammer, 1993; Balciunas et al., 2013). In 2005, Cotter recommended another order, with two classes in this idea: class I (lantibiotics) and class II (different peptides without lanthionine). High molecular weight thermolabile peptides were prohibited from the bacteriocin classes and were independently categorized as bacteriolysis. The authors additionally recommended that class IV of the classification should be excluded (Cotter et al., 2005). In 2006, Drider et al. (2006) at long last separated bacteriocins into three fundamental classes, by utilizing their hereditary and biochemical qualities.

### Class I

Individuals from class I bacteriocins, additionally called lantibiotics, are small (19–38 amino acid residues) and thermostable peptides. Past studies show that the molecular structure of lanthionine or β might be responsible for thermostability (Balciunas et al., 2013). The most common example of the Class I group is nisin. A few strains of *Lactococcus lactis* subsp. lactis can naturally produce bacteriocin, and it contains 34 amino acid residues in its molecular structure. The two variations of nisin are nisin An and nisin Z. The two have a similar molecular pattern excluding one amino acid, yet show similar antimicrobial activity. Additionally, there is another variation of nisin obtained from *Streptococcus uberis* and named nisin U with 78% similarity to nisin A (Diep and Nes, 2002; Balciunas et al., 2013).

Many scientists showed that nisin displays a wide-range spectrum of antimicrobial impacts on different microbes and LAB species including *Listeria monocytogenes*, *Staphylococcus aureus*, and *Bacillus cereus*. In its method of activity system, nisin influences the target cell wall and membrane utilizing a double activity component, causing pore formation, the outflow of necessary compounds (K^+^ particle, amino acids, and ATP) through the pores, penetrability changes, and finally the target cell lysis (Balciunas et al., 2013). Nisin can be utilized in various technological applications due to its broad range of antimicrobial action. In 1969 Food and Agriculture Organization/World Health Organization (FAO/WHO) approved nisin as the only bacteriocin which is safe for food application. Nisin is also used as a bio-preservative ingredient with the number E234 in European Union countries (Table 1) (Balciunas et al., 2013).

**TABLE 1:** Example of bacteriocin classification.

Sl.No.	Classification	Sub- categories	Examples	Characteristics	References
1	Class I (lantibiotics)	Type A (linear molecules) and Type B (globular molecules)	Nisin, subtilisin, epidermine, and mersacidin	Lanthionine or peptides containing β-lanthionine	Drider et al. (2006) and Balciunas et al. (2013)
2	Class II	Subclass IIa (antilisterial-pediocine bacteriocin type) Subclass IIb (composed of two peptides) Subclass IIc (other bacteriocins)	Pediocin, enterocin, sakacin. plantaricin, lactacin, and lactococcin	Heterogeneous class of small thermostable peptides	
3	Class III	—	Helvecitin J and millericin B	Large thermolabile peptides	

### Class II

Class II bacteriocins comprise huge and different categories of ribosomally synthesized antimicrobial peptides. As Class II bacteriocins do not have post-translational modifications in the peptide chain, class II bacteriocins have simpler structures than lantibiotics, for example, lanthionine or β-lanthionine. This class incorporates small thermostable (<10 kDa) peptides with an amphiphilic helical structure. The cytoplasmic membrane insertion at the target cell is due to the structural conformation of class II. This results in depolarization of the membrane and cell lysis.

Class II bacteriocins can be categorized into three subclasses: subclass II-A, subclass II-B, and subclass II-C (Balciunas et al., 2013).

#### Subclass II-A

High antibacterial activity is an important characteristic feature of the members of subclass II-A. There are 37–48 amino acid residues present in the molecular structure of these bacteriocins. A pleated sheet configuration is present in the N-terminal part of the compound, and the C-terminal portion contains a couple of α-helices. In the method of action, a bacteriocin from the subclass II-A falls into the cell membrane of the objective cell by the C-terminus. As a result, pore formation enhances and causes dissemination of proton motive force that causes high ATP utilization and finally causes death. Some examples of subclass II-A are pediocin, enterocin, and sakacin (Balciunas et al., 2013).

#### Subclass II-B

Heterodimeric bacteriocins are a part of subclass II-B which comprises two peptides. This subclass member should meet the following criteria:

• Full antimicrobial action needs both peptides and the singular peptides to show almost no action
• Immunity can be obtained by utilizing one immunity protein
• Two sequential structural genes of bacteriocin encode a single immunity gene, and individual peptides are incorporated within the genetic system of the bacteriocin.

The first bacteriocin discovered in this group is *Lactococcin G*. The antimicrobial activity of *Lactococcin G* relies upon both α-and β-peptides. Some other important examples of two-peptide bacteriocins include plantaricin and lactacin F. Their system of activity includes membrane potential dissipation and a reduction in the intracellular ATP concentration (Cintas et al., 2001; Diep and Nes, 2002; Riley and Wertz, 2002; Balciunas et al., 2013). For obtaining the entire antimicrobial activity, the presence of the two peptides are necessary, although in some cases the individual peptide can act as a residual peptide, and the effect is subtle in this case.

#### Subclass II-C

The features of this subclass of bacteriocins are that they are circular, and there is a presence of a covalent bond between the C and N terminal, which results in the peptides having a tail cyclic shape. The fundamental agent and the most concentrated illustration of this subclass are AS-48 from E*scherichia faecalis* as the most common example of this subclass. AS-48 mode of action includes the permeabilization of the cytoplasmic membrane of the target cells, bringing about the dissipation of the proton motive force, and finally causing cell lysis (Balciunas et al., 2013).

### Class III

This class incorporates huge thermolabile bacteriocins within more than 30 kDa molecular weight. Complicated action and protein structure providing the distinguished mode of action from other bacteriocins, causing cell wall lysis of the target microbes form one of the key factors of the class III group. The N-terminal part acts as an endopeptidase for this mechanism, and the target cell is recognized by the C-terminal (Balciunas et al., 2013).

## Bacteriocin synthesis and its transport

It was observed that bacteriocin-producing genes are typically found in the operon cluster. For the production of lantibiotics, homologous genes are present in the sequenced lantibiotic operons. Operons belonging to Class Ia lantibiotic were mostly characterized, whereas the gene cluster for mersadicin, a Class Ib lantibiotic, was explicated recently. Many genes present in the cluster are able to transcribe proteins which are analogous to Class Ia. Most of the bacteriocin-producing genes were either located on chromosomes, or encrypted in plasmids or transposons. Some structural proteins are able to process the transportation of bacteriocins across the membrane and confabulate the host immunity to the producers. Both the lantibiotic and non-lantibiotic bacteriocin-encoding genes possess a similarity in structure and transport and regulatory mechanisms. However, all the bacteriocins belonging to the different classes are ribosomally synthesized with an exception of Class I, which is post-translationally modified.

Translocation of Class I and II bacteriocins is carried out through the ABC transporter system, whereas few class II bacteriocins are manifested *via* sec-dependent systems. ABC transporter-dependent bacteriocins are divided into two significant groups, one being bacteriocins with a double glycine leader, and the other is bacteriocins with a different leader, but not a sec-leader. Several studies confirmed that double-glycine leader bacteriocins are usually present in Class II bacteriocins, including some lantibiotics (Håvarstein et al., 1994; Nes et al., 1996).

The secretion of these bacteriocins is mediated by the unique form of ABC transporters with a 150-amino acid residue N-terminal leader sequence that exerts proteolytic activity to the double-glycine leader in order to activate bacteriocins. This secretion process is triggered by a specific accessory protein (Franke et al., 1996).

**FIGURE 1:**
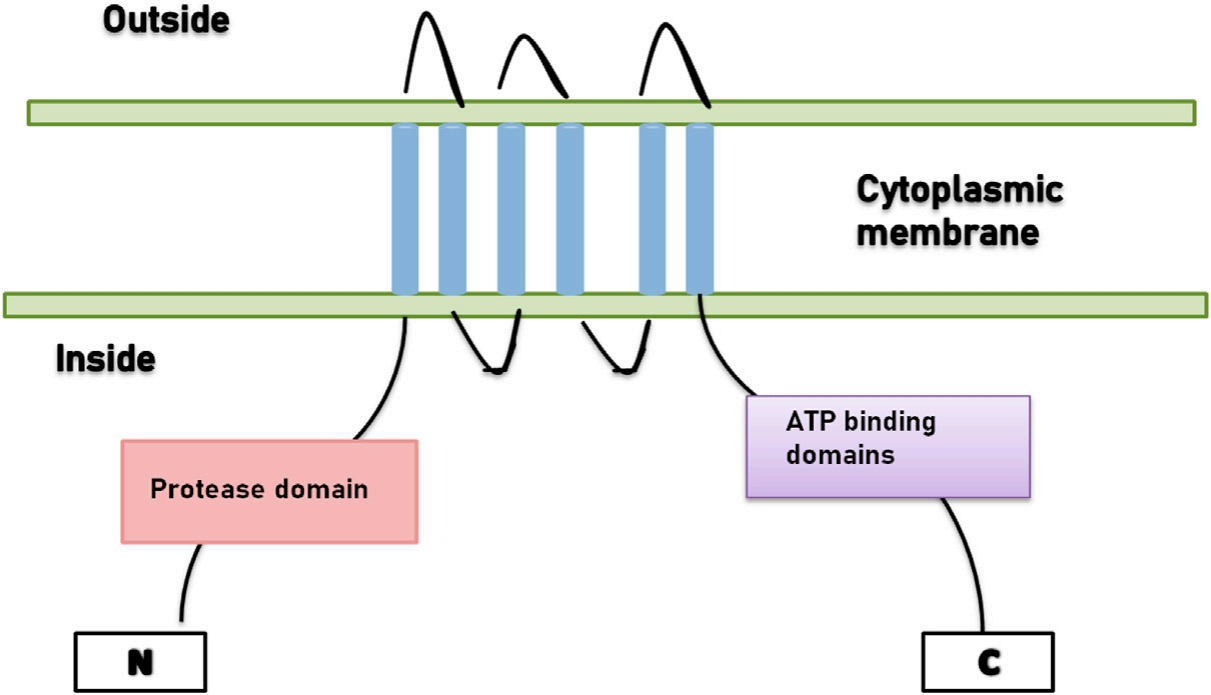
ABC-transporter with N-terminal domain.

Lantibiotics with a distinct leader, which is secreted by the ABC transporter, do not acquire N-terminal proteolytic activity. A diligent protease is accountable for the removal of the leader sequence, for example, NisP is the protease present in the nisin system (Figure 1).

## Genetics of bacteriocin

### Class I bacteriocins

Lantibiotics are the class I bacteriocin that is manifested as pre-pro-peptides with N-terminal leader sequence and C-terminal propeptide which get post-translationally modified. The biosynthesis of lantibiotics is usually pioneered through the enzymatic dehydration of serine and threonine residues present in the propeptide parts, in order to yield unconventional amino acids 2,3-dehydroalanine (Dha) and 2,3-dehydrobutyrine (Dhb). From neighboring cysteine residues, the thiol group is appended to these dehydroamino acids’ emerging attributes to the lanthionine (from Dha) and methyllanthionine (from Dhb) residues. The three-dimensional structure of the peptides is determined by the thioether-based intramolecular rings in order to exhibit the biological activities. The leader peptide contains a particular site for substrate recognition for the modification enzymes. The amalgamation of nisin leader is prior to the synthesis of pneumococcin by the nisin modification enzymes in one of the example of leader peptide (Majchrzykiewicz et al., 2010).

The production of lantibiotics generally depends on the Gram-positive bacteria. The genetic determinants for the synthesis of lantibiotics are not only present in genera and phyla, but they are also tracked down on chromosome or mobile elements, in the form of plasmids and transposons. Gene clusters contains several genes which are responsible for the peptide structure determination (lanA), modification (lanB, lanC, lanM, labKC, lanL and lanD), proteolytic processing (lanP and lanT), transport (lanT), immunity (lanI and lanFEG), and biosynthesis regulation (lanK, lanR, and lanQ) (Chatterjee et al., 2005; Willey and van der Donk, 2007; Bierbaum and Sahl, 2009).

It was observed that the LanM enzyme in the case of cyanobacterium Prochlorococcus can modify the profusion of LanA precursor peptides which are encoded everywhere on the chromosome (Bo et al., 2010). Recently, the classification of lantibiotics was carried out depending on their modification machinery and antibiotic activity. Lantibiotics exhibiting antimicrobial activity come under type I and type II, whereas lantibiotics with little antibiotic activity are considered under type III (Willey and van der Donk, 2007).

Several distinct biosynthetic mechanisms are involved in the biosynthesis of the ring structure. The two enzymes LanB and LanC are required to synthesize lanthionines, which are mainly found in the gene cluster of type I lantibiotics (Chatterjee et al., 2005). The Ser and Thr residues of the C-terminal of the propeptide sequence or the core propeptide are dehydrated by the LanB enzymes. The cyclization reactions in the formation of thioether structures are catalyzed by the LanC enzyme. In the structure of NisC, zinc ion coordinates to the enzyme and activates thiolate (Bierbaum and Sahl, 2009). A single enzyme LanM is required to form the thioether bridge of the type-II lantibiotics and catalyzes both the reactions (Chatterjee et al., 2005; Bierbaum and Sahl, 2009).

In *in vitro* modification assays, several LanM enzymes are activated, followed by ATP-dependent phosphorylation which mediates the dehydration of hydroxy amino acids (McClerren et al., 2006; Bo et al., 2010).

The LanD enzyme is accountable for the oxidation of C-terminal Cys residue of epidermin, along with its analogs, and also in mersacidin (Sit et al., 2011). A novel modified amino acid labionin, which is a quaternary α,α-disubstituted amino acid, is present in labyrinthopeptins and is modified in a different biosynthetic mechanism involving GTP-dependent phosphorylation through LabKC enzyme (Müller et al., 2010; Müller et al., 2011).

The antibiotic action of the LabKC enzyme has not been reported yet, whereas RamC, which has similarity with the LabKC enzyme, is able to modify the type III lantibiotic SapB.

The biosynthesis of lantibiotics is growth-phase dependent and is regulated *via* the dual unit signal transduction systems. A membrane-bound sensor and a histidine protein kinase (HPK) are the two protein components of the intracellular signaling system that regulate the environmental signals. The adaptive response due to the change of genetic expression is synchronized by the cytoplasmic response regulator (RR). The His residue present in C-terminal cytoplasmic domain is auto-phosphorylated after receiving the external signals by the HPK, followed by the transfer of the conserved Asp of the intracellular transcriptional activator i.e., RR. The genes encoding both HPKs (LanK) and RRs (LanR) are present in the gene cluster of nisin, mersacidin, SA-FF22, and subtilin (Klein et al., 1993; Engelke et al., 1994; McLaughlin et al., 1999; Altena et al., 2000).

Several studies on nisin and subtilin substantiate the presence of these genes for the production of bacteriocins. The RR is promoter of lanA, i.e., the structural gene of lantibiotics and the fully modified nisin are able to autoregulate their own biosynthesis through the quorum-sensing peptides nisA and nisB (Kleerebezem et al., 1997).

The ribosomally synthesized, post-transcriptionally modified peptides bacteriocins have specific operon clusters. The genes present in the operon cluster are accountable for the indemnity of the bacteriocins. These genes are present in the chromosome and are associated with transposons and plasmids (Deegan et al., 2006). The preliminary synthesized prepeptides with N-terminal domain are biologically inactive in nature. Before transportation the bacteriocin gene cluster encoded proteins and amino acids modify the pre-peptide. For instance, the thioether cross-linker lanthionines (Lans) and methyl lanthionines (MeLans), along with 2.3-didehydroalanine (Dha) and (2).-2-3-didehydrobutyrine (Dhb) amino acids, are introduced through the stereo-selective intermolecular addition of cystine after the successive dehydration of serine and threonine residues (Cleveland et al., 2001). The production of specific immunity proteins protects the bacteriocin-producing strains from their own toxin effects. Genes encoding immunity proteins are homologous to the structural and processing genes of bacteriocins, and are located at the same operon system. The dual immunity system of bacteriocins is dependent on specific immunity LanI and multicomponent ABC transporter (LanEFG). The LanI is usually remains attached to the outer surface of the cytoplasmic membrane of producer cells, in order to provide protection by preventing pore formation, and retains the bacteriocin concentration in membrane up to the pivotal level (Todorov and Dicks, 2004; Todorov and Dicks, 2005).

### Class II bacteriocins

Class II bacteriocins are small and heat-stable but do not comprise Lan residues. Class II bacteriocins consist of four subclasses.

#### Subclass IIa

Class IIa has been useful in food preservation since it contains pediocin-like Listeria active peptides, with examples including pediocin PA1 and leucocin A (Mokoena, 2017). As a prebacteriocin, which has an N-terminal leader sequence to keep the peptide inactive, Class IIa bacteriocin is initially produced *via* ribosomal synthesis. In all, 15 to 30 residues make up the leaders, the majority of which are double-glycine residues located upstream of the cleavage site. The leader is thought to act as a signal sequence for bacteriocins to be processed and secreted by a special system made up of an ABC transporter and an auxiliary protein. The N-terminal transmembrane domain and the C-terminal ATP-binding domain of the ABC-transporter protein are integrated into the membrane bilayer. The leader peptide can be cleaved at the double-glycine motif by the N-terminal region (Zhang et al., 2022).

A quorum-sensing (QS) system, which comprises an inducing peptide, membrane-associated histidine protein kinase (HPK), and a cytoplasmic response regulator, normally controls the synthesis of class IIa bacteriocins (RR). The inciting peptide is created as a pre-peptide, with an N-terminal leader sequence that the ABC-transporter cleaves upon secretion. The co-expression of immunity proteins allows the bacteriocin-producing bacteria to avoid being killed by their bacteriocins. The length and sequence diversity of the immunity proteins for the class IIa bacteriocins range from 81 to 115 amino acids (Martin-Visscher et al., 2008).

The precise recognition of their associated bacteriocins is carried out by the C-terminal region. Possession of a plasmid is frequently linked to the production of class IIa bacteriocins. Enteriocin A, divercin V41, sakacin P, and canobacteriocin B2 are examples of biosynthetic gene clusters that may occasionally be found in the chromosome. This class’s genomic structure exhibits a great deal of conservation. ABC-transporters and their supporting proteins are often encoded by an operon that is part of the gene cluster that codes for class IIa bacteriocins.

#### Subclass IIb

Class IIb bacteriocins are created as precursor peptides with leader peptides, which are N-terminal extensions that are cut off during maturation. Currently known as class IIb bacteriocins, all have a double-glycine type leader. The inactive pre-peptide is broken down by the ATP-binding cassette (ABC) transporter and an accessory protein, which results in the concurrent export of the mature bacteriocin across the cytoplasmic membrane. The auxiliary protein may have a role in bacteriocin immunity or be necessary for bacteriocin secretion (Zhang et al., 2022). A combination of two distinctive peptides makes up class IIb. These peptides appear to have no or very little sequence in common with one another, and they have little or no activity (Mokoena, 2017).

Subclass IIb bacteriocin production is frequently controlled by a three-part regulation scheme. The matching HK detects the inducing peptide as a cell density indicator, activating the RR, which then stimulates the expression of all operons required for bacteriocin production, transport, and regulation (Zhang et al., 2022). Five to eight genes typically make up a gene set for the synthesis of class IIb bacteriocins. Among these are two genes that produce bacteriocins, which are closely related to the neighboring gene that produces the immunity protein. The three-component regulatory system genes may be found upstream or downstream of the genes encoding bacteriocin structural components. An ABC transporter complex is encoded by two genes in the majority of class IIb gene clusters (Cebrián et al., 2015).

#### Subclass IIc

Circular bacteriocins are biosynthesized through the cleavage of the leader, circularization, and exporting of the mature bacteriocin. Leader cleavage is thought to be the initial stage of maturation and a prerequisite for additional processing to produce mature bacteriocins. There is no sequence resemblance among the leader peptides, which range in size from 2 to 35 amino acids, and it is yet unknown how the leaders work. The leader of circular bacteriocins does not typically cleave at the double-glycine site, in contrast to class IIa and IIb bacteriocins, which typically do (Zhang et al., 2022).

All circular bacteriocins have ligation sites that are situated inside of a helical shape that is primarily made up of hydrophobic residue stretches. It was proposed that the circularization reaction requires a hydrophobic environment. The effectiveness of the circularization process depends on the characteristics of both the N- and C-terminal residues. Immunity to circular bacteriocins has been linked to several proteins. The specific immunity proteins for AS-48, gassericin A, and carnocyclin A are As-48D1, GaaI, and CclI, respectively. These immune proteins have one or two transmembrane domains, are tiny (49–56 amino acids), cationic (high pI), and may be found in the cell membrane. Moreover, the transportation system for class IIc is more complex, compared to other subclass II bacteriocins. There are accessory operons that encode an ABC transporter complex, consisting of a permease, an ATPase, and an extracellular protein.

#### Subclass IId

The biosynthetic mechanism of the majority of the leaderless bacteriocins still needs to be researched in more depth. Other general bacteriocins’ leader sequences are crucial for recognition by transporters. Additionally, until it is time for secretion, the leader sequences keep the precursor peptides dormant during biosynthesis inside the host. The non-pediocin liner bacteriocins are produced as physiologically inactive pre-peptides with an N-terminal leader peptide, just like class IIa and IIb bacteriocins. Following pre-peptide synthesis, a specific membrane protein from the ATP-binding cassette transporter family cleaves the N-terminal leader sequence at the double glycine site. The majority of the leaderless bacteriocins have gene clusters that have been found. Bacteriocin structural genes frequently share close relationships with genes involved in immunity and transport. Leader-containing bacteriocins require an auxiliary protein to mediate bacteriocin secretion, in addition to the associated ABC transporter. For the transportation of leaderless bacteriocins, such an auxiliary protein is not necessary. A host-encoded formylase that occurs outside of the biosynthetic gene cluster may be responsible for performing the N-terminal formylation of leaderless bacteriocins because formylase synthesis-related genes were not discovered close to the bacteriocin structural gene. Most leaderless bacteriocin regulation is linked to environmental factors.

## Omics in bacteriocin production and regulation

In order to study microbes in detail, genomic information is one of the essential factors which usually provide persistent linkage to other organisms. For example, fully sequenced genomes of various lactic acid bacteria species are available, which are useful in assembling draft genomes of unknown bacterial strains. Excavation of genomic information stipulates the presence of specific attributes in the microbes. Significant genetic codes and specific pathways deduce potential products from the microbes. Comparative genomic analysis between genetic pathways of LAB and strain under consideration accentuates the specificity of microbial function (Makarova et al., 2006). Extraction of genomic data is able to propound the functional characteristics of the target microbial strains. For instance, genomic data analysis of *Lactobacillus ruminis* disseminates the presence of operative flagellar framework in 45 flagellar gene conformation (Neville et al., 2012). The resilient flagellar framework indicates mortality and proinflammatory propensity of the *L. ruminis*. Several gene clusters encoding significant mucus binding pili are present in LAB, resulting in the adherence of *L. rhamnosus* into the intestinal mucosa (Kankainen et al., 2009). Along with this bacteriocin-producing gene clusters are also detected. Several software tools for secondary metabolites and bacteriocin detection analyze the genome of LAB, followed by the detection of biosynthetic gene clusters through anti-SMASH, PRISM, and GRAPE software. The unblocking of the genomic data set of LAB revels the capability of producing diverse antimicrobial peptides. Several powerful analysis tools are capable for analyzing the functional potentially of the bacterial genomic data set, in order to screen out the unique antimicrobial compounds like bacteriocins. Around twenty LAB genomes were apprised for the bacteriocin-producing genes with few recognized operons for bacteriocin characterization. The third-generation sequencers like Minion and Sequel II are able to resolve the native issues associated with second-generation sequencers for utilizing genomic information (Rhoads and Au, 2015; Lu et al., 2016).

This third generation-sequencer is particularly a GC-biased fragmentation and amplification, leading to sequence repetition and genome rearrangement. Antibiotics resistance and bacteriocin production genes are usually present at the transposable elements. The presence of inverted repeats during the genome replication plays a crucial role in the transfer of genomic information between different species (El Kafsi et al., 2017). This mechanism appears as an important aspect in determining specific traits of LAB in discovering novel bacteriocins. The characterization of gene clusters encoding antibiotic resistance along with translocation of patho-adaptive features in between mutualistic pathogenic bacteria present in microbiota has been facilitated by the analyzing ability of more than 20 kb (Proença et al., 2017).

## Mode of action of bacteriocin

Depending on the bacteriocin type, the mode of action is usually determined. The primary receptors of bacteriocins are located in the cytoplasm and they are lipid molecules which are anionic in nature. The efflux within the ions and molecules outside the cells is caused due to the pore formation because of the bacteriocin binding, which results in damage and cell lysis. Pore formation in the case of the lantibiotics is dependent on lipid II and peptidoglycan receptors which also behave like docking molecules. The uniqueness of class II bacteriocins is determined by receptor molecules present within the cell membrane (Venema et al., 1995a; Venema et al., 1995b). The pore formation of different bacteriocins follow various models, like Class I bacteriocins having a wedge-like model, whereas Class II bacteriocins follow a barrel stave or carpet-like model; the bacteriocins are located in parallel on the membrane surface and also cause disruption of the cell membrane (Moll et al., 1999). The bacteriocin widely used as a food preservative is nisin, which acts as a surface active molecule with cationic detergent. The adsorption of bacteriocin takes place across the cell membrane, the lipid II component binds to the bacteriocin, the poration complex is stabilized, and sulfhydryl groups is degraded, finally causing disruption of the cell (Bruno et al., 1992). The lipid II molecules are also isolated, and they damage the repair mechanism of the cells of the bacteria by preventing cell wall biosynthesis. The lipid II molecule interaction of class II lantibiotics like mersacidin leads to cell wall biosynthesis inhibition. The composition of lacticin 3147, which belongs to lantibiotics, consists of both lac 1 and lac 2 component systems (McAuliffe et al., 1998). The pore formation requires its synergistic activity within the membrane of the targeted cell. Peptide A1 is mainly utilized for interaction of lacticin with the membrane of the cell, which is followed by lipid II component binding with the cell wall. As a result, there is alteration within the A1 peptide, leading to formation of the affinity binding site for A2, which is the second component of the bacteriocin; thus, it affects the pore formation of the cell membrane (Lawton et al., 2007). In case of pediocin, which belongs to subclass IIa, binding takes place between the IIAB, IIC, and IID subunits that are part of the mannose phosphotransferase system (M-PTS). Moreover, IIC and IID subunits are recognized by the bacterion, and IIc behaves like a receptor. Furthermore, the bacteria infuses itself within the cell membrane, leading to pore formation, and finally resulting in efflux of ions and molecules (Héchard and Sahl, 2002).

## Selection criteria of bacteriocin for application as a food preservative

For a bacteriocin to be used as a food preservative, certain qualities are to be checked:

1. The bacteriocin should be safe for human consumption and it should also be safe for the human intestinal microflora.
2. The bacterion should have a broad antimicrobial spectrum of activity against food spoilage microbes.
3. It should be enzyme-resistant with the food matrix.
4. It should have stability to high temperatures, and a broad pH range and salt concentration for a broad range of food processes.

Bacteriocin safety is evaluated by performing several assays, including cytotoxicity assays within the eukaryotic cell lines (Murinda et al., 2003; Weyermann et al., 2005) and their capability to induce apoptosis, hemolytic action, inhibition of growth, *in vitro* cross-resistance and chronic toxicity, impairment in reproduction, and sensitization in animal models; all of these should be completely removed (Vaucher et al., 2011). The total elimination of cytotoxicity is not possible, but cytotoxic concentration is found to be much higher than the minimum inhibitory concentration which is required for food spoilage. Bacteriocins which are obtained from LAB are generally safe for application, except enterococcal cytolysin which has broad cytotoxic action (Cox et al., 2005). There is usage of bacteriocinogenic strains as starter cultures for various fermented food processes that are not genetically modified. Bacteriocins are given GRAS status if it has been previously used as a food preservative or approved by the FDA as safe for usage. The utilization of bacteriocin delivering cultures for *in situ* development of bacteriocin are liked in instances of fermented food sources lessening the expense needed for the purification of bacteriocins. Favorable innovative characteristics like high acid and flavor development by the LAB strains can be utilized as sole starter cultures for fermentation, enjoying the double benefit of preservation and fermentation.

If the bacterial strains are not suitable for fermentation processes, they can be utilized as adjunct cultures alongside primary fermenting cultures. Thus they have no interference with the action of the fermenting bacterial strain. The usage of bacteriocin-producing strains for the preservation of non-fermented foods is carried out if they do not impart flavors or any bad odor, and also if the organoleptic quality of the food remains unaffected. Bacteriocin-producing starter cultures for application as food additives must be of GRAS status, as given in the guidelines of food safety regulations. For usage of the purified bacteriocins, they should follow the guidelines for the safety evaluation of novel preservatives provided by the U.S. FDA. Moreover, the bacteriocin should be characterized and chemical identification should also be carried out. Moreover, the efficiency and the usage of the bacteriocin should be characterized, with its toxicology as well as pharmacodynamics of the molecules prior to digestion. Further report should be given on the manufacturing and standardized assays, for approval to be used as food additives (Johnson et al., 2018).

GRAS status is usually given to bacteriocinogenic cultures, due to their presence within fermented foods and also due to their consumption by humans over centuries. But for their application as a food preservative for non-fermented foods or usage as a food additive, they still require approval from the FDA. The safety of this naturally occurring is to be evaluated further before their usage (Alvarez-Sieiro et al., 2016).

## Application of bacteriocins

The constant adverse effects on human health due to consumption of chemical additives have led to consumers being more aware of the need for natural preservatives which have no harmful effects on human health. The search for an alternative to chemical additives with a long shelf life has led to extensive research work in this field of developing new natural preservatives. An alternative to these chemical preservatives which is natural and non-harmful in nature is bacteriocins obtained from lactic acid bacteria (LAB). These bacteriocins can also be utilized as food biopreservatives (Table 2). This application of bacteriocins as biopreservatives can be carried out by:

• Inoculation of foods with bacteriocins obtained from LAB
• Bacteriocins purified or semi-purified before use as food additives.
• Addition of bacteriocins to products which are fermented beforehand and utilizing them in various food processes.

**TABLE 2:** Examples of bacteriocin as a food preservative.

Name of bacteriocin	Application	Advantage of using them	Gene	Reference
Nisin A	Nisin is incorporated within the meat binding system	Nisin addition prevents the growth of unwanted bacteria within the meat products	*nisZBTCIPRKFEG* gene cluster	Cutter and Siragusa (1998)
Enterocin 4	*Enterococcus faecalis INIA4* is used as an enterocin producer which is utilized as a starter culture for production of Manchego cheese	*Enterococcus faecalis INIA4 inhibits t*he growth of *L. monocytogenes Ohio* but not *L. monocytogenes Scott A*	*ej97A*	Nuñez et al. (1997)
Pediocin AcH	Pediocin obtained from *Lactobacillus plantarum WHE 92* is spread on the surface of Munster cheese at the beginning of the ripening phase	The spray prevent the overgrowth of *L. monocytogenes*, and it can be used for antilisterial treatment	*papA*	Ennahar et al. (1996)
Pediocin	Operonis expressed in *Saccharomyces cerevisiae*	It is mainly used in the preservation of wine and baked products	*ped* genes	Schoeman et al. (1999)
Pediocin PA-1	It is mainly used as a starter culture in the sausage fermentation	It mainly prevents the growth of *L. monocytogenes*	*pedA*	Foegeding et al. (1992)
Leucocin A	It is used to control meat spoilage	Inoculation of vacuum-packed beef increases the shelf life of *Lactobacillus sake* upto 9 weeks	*lcaCD *	Leisner et al. (1996)
Lactocin 705	It mainly prevents the growth of *L. monocytogenes* in ground beef	It mainly prevents the growth of *L. monocytogenes* in ground beef		Vignolo et al. (1996)
Piscicolin 126	It mainly used in controlling *L. monocytogenes* in devilled ham paste	It is more effective than other bacteriocins		Jack et al. (1996)
Linocin M-18	Their starter cultures are used for the production of smear cheese	It causes 2log reduction of *L. monocytogenes*		Eppert et al. (1997)
Enterocin	It is added to inoculated ham, pork, cheese, chicken, and sausage	It helps in controlled growth of *L. monocytogenes* under certain conditions	*entA, entI,* and *entF *	Aymerich et al. (2000)

The effectiveness of bacteriocins in food applications should be carefully examined, though the application of nisin in various food processes has been carefully examined (Abee et al., 1995; Delves-Broughton et al., 1996).

Bacteriocin activity is greatly influenced by both physical conditions and the chemical composition of food. The solubility of nisin increases by 228 times in pH 2 compared with pH 8 (Liu and Hansen, 1990). Many researchers utilize bacteriocins in starter cultures, as LAB are utilized in food fermentation processes mainly as starter cultures. Many studies also utilize natural bacteriocin-producing microorganisms such as *Pediococcus acidilactici and Enterococcus faecalis* (Campanini et al., 1993; Nuñez et al., 1997)*.*

It was observed that cheese, when treated with *Enterococcus faecalis*-producing bacteriocin, there was a decrease by 6 logs in 7 days, but the survival rate in commercially producing starter cultures remains unaffected. In one study, it was observed that both pediocin PA-1 and nisin were both effective and safe expressed in *Lactobacillus lactis* (Horn et al., 1999). The transformed cells can be applied in improving the safety of food and also to reduce the resistant organisms, as the cells produce 11.8% pediocin levels as compared to the control. Pediocin PA-1 is also utilized in the preservation of bread, wine, and other food products (Schoeman et al., 1999).

## Application in meat products

*L. monocytogenes* is a rod-shaped gram-positive, non-spore-forming facultatively anerobic, generally found naturally. Its optimal growth occurs at a pH ranging between 4.1 and 9.6 and a temperature ranging between 0 and 45°C. In addition, it is desiccation-resistant; furthermore, it can develop at aw values as low as 0.90. The omnipresent nature of *L. monocytogenes*, its strength and ability to grow at freezing temperatures and anaerobic environment make it an risk to the safety of food sources. It is viewed as a significant food safety issue on the grounds that it can cause disease and death. The United States government has the most rigid strategy in regards to *L. monocytogenes;* furthermore, they have set no tolerance of *L. monocytogenes* in packaged food sources (Jay et al., 2005). It has been recognized in various food sources and in a few foodborne outbreaks, for example, turkey franks. Many investigations have been completed to control *L. monocytogenes* in meat items since it is normal inside slaughterhouse and meat-pressing conditions; also, it has been isolated in raw meat, and cooked and ready-to-eat meat items (Table 3).

**TABLE 3:** Bacteriocins used in meat products.

Meat products	Bacteria producing them	References
Dry fermented sausages	*Staphylococcus xylosus DD-34, Lahti and others 2001 Pediococcus acidilactici PA-2, Lactobacillus bavaricus MI-401, and Lactobacillus sake CTC494*	Lahti et al. (2001)
Chicken summer sausages	*Pediococcus acidilactici*	Baccus-Taylor et al. (1993)
Salami	*Lactobacillus plantarum MCS*	Campanini et al. (1993)
Brazilian sausage	*Lactobacillus sake 2a*	Liserre et al. (2002)
Turkey summer sausage	*Pediococcus acidilactici JBL 1095*	Luchansky et al. (1992)
Wieners	*Pediococcus acidilactici JBL 1095*	Degnan et al. (1992)
Beef cubes which are minimally heat-treated	*Lactobacillus bavaricus MN Winkowski and others 1993 wieners Pediococcus acidilactici JBL*	Winkowski et al. (1993)
Frankfurters	*Pediococcus acidilactici JD1-23*	Berry et al. (1991)
Minced meat and pork into casings	*Lactobacillus sake Lb 706*	Schillinger et al. (1991)

*Lactobacillus* spp are easily found in meat, and hence bacteriocins produced by LAB are commonly isolated and utilized for various purposes. Bacteriocins are utilized in various food processes and systems, but they are not alone used as a food additive. Bacteriocins isolated from LAB obtained from various food sources might not be effective in all food systems. Under suitable conditions, certain bacteriocins have the ability to become a potential food preservative, such as nisin which can be effective in meat systems. Nitrates were previously utilized in the preservation of meat as nitrates prevent clostridial growth in meat, but it results in the presence of nitrates in meat which was becoming a safety hazard, so the industry is looking for various non-harmful alternatives. Nisin or its mix with lower levels of nitrate can hinder the development of *Clostridium* (Rayman et al., 1981; Rayman et al., 1983).

However, studies show that nisin’s effectiveness when applied to meats is not great, due to its high pH and ineffectiveness in its uniform distribution, and also due to the interference of the phospholipid components of meat (Rayman et al., 1983; Chung et al., 1989; Valdés-Stauber and Scherer, 1994).

A study has shown that nisin is inactivated by glutathione in a response catalyzed by glutathione S-transferase (Altena et al., 2000). Glutathione is found in raw meat, and the glutathione reaction significantly decreases the action of nisin. Other studies show that nisin can be utilized in meat under specific circumstances. Bacteriocin can increase the shelf life of sausages as well. A study showed that the usage of fat content and phosphate emulsifiers increases nisin affectivity in sausages (Davies et al., 1999). It was seen that nisin effectivity was inversely proportional to the fat content of the meat. Studies show that when nisin is combined with lactic acid, there is an increase in effectiveness against Gram-negative bacteria (Ariyapitipun et al., 1999; Ariyapitipun et al., 2000).

Nisin can also be used in the cold meat binding system, as it is effective against *Brochothrix thermosphacta* (Cutter and Siragusa, 1998). As there are problems associated with direct application of nisin in raw meats, other bacteriocins are also under examination. Leucocin A, enterocins, sakacins, and the carnobactericins A and B are utilized in increasing the shelf life of raw meat. Pediocin PA-1 obtained from *P. acidilactici* is observed to diminish target organisms as they contain an identical amino acid sequence to AcH, though this is yet to gain approval to be used as a food preservative in the United States (Nielsen et al., 1990).

### Seafoods

The viability of bacteriocins and protective cultures to control the development of *L. monocytogenes* in vacuum-pressed cold smoked salmon has been shown by a few scientists. The inhibitory impact of sakacin P was analyzed, as well as L. sake cultures (sakacin P producer) against *L. monocytogenes* in cold smoked salmon. The vacuum-bundled salmon samples were incubated at 10°C for 4 weeks (Katla et al., 2001). Sakacin P affects the development of *L. monocytogenes*, while cultures of *L. sake* made a bacteriostatic difference. At the point at which L. sake culture was added to salmon along with sakacin P, a bactericidal impact against *L. monocytogene*s was noticed. Nilsson et al. (1999) showed that a non bacteriocin-producing strains of *C. piscicola* was basically as effective as a bacteriocin-delivering strain of *C. piscicola* in the inhibition of *L. monocytogenes* in vacuum-pressed cold-smoked salmon. They recommended that the growth inhibition of *C. piscicola* that brought about essential nutrients was depleted.

The inhibitory impact of nisin in combination with carbon dioxide and low temperature on the endurance of *L. monocytogenes* in cool smoked salmon has been examined (Nilsson et al., 1997). Growth of *L. monocytogenes* within vacuum packs was not hindered by nisin addition (500 or 1000 IU/g) to salmon when inoculation with *L. monocytogenes* was carried out at 5°C storage. It was observed in the vacuum-packed salmon that there was an increase in the number of *L. monocytogene*s to 108 CFU/g after 8 days, whereas when cold salmon were packed with carbon dioxide, the number of *L. monocytogenes* after 27 days was 106 CFU/g, i.e., there was an 8-day lag phase for *L. monocytogenes.* Now when nisin was added to cold smoked salmon, there was a decrease of *L. monocytogenes* from 1- to 2-log 10. It was also followed by a lag phase of 8 and 20 days using 500 and 1000 IU nisin/g respectively. It was observed in both that the nisin concentrations *L. monocytogenes* levels were below 103 CFU/g after 27 days.

Brined shrimp shelf life is enhanced by addition of sorbic and benzoic acids. But the harmful effects due to the usage of organic acids have led researchers to find an alternative which led to the usage of naturally producing bacteriocins for preservation. A study evaluated the efficacy of nisin Z and carnocin UI49, and the development of bavarcin A on enhancing the shelf life of brined shrimp (Einarsson and Lauzon, 1995). It was observed that carnocin did not enhance the shelf life which was 10 days when compared to the control, whereas bavaricin increased the shelf life to 16 days, and nisin Z enhanced the shelf life up to 31 days. But it was seen that the benzoate-sorbate solution increased the shelf life up to 59 days, so we can say that it improved maximum shelf life when compared with other bacteriocins.

### Dairy products

The documentation of *L. monocytogenes* is mainly carried out due to its numerous outbreaks related to dairy products including pasteurized milk and cheese (Linnan et al., 1988). It has also been found that nisin is effective in dairy products against *L. monocytogenes*. It was also observed that there was decrease in the amount of *L. monocytogenes* upto 1-log10 cycle when inoculated with nisin-resistant strain with cottage cheese at a pH ranging between 4.6 and 4.7 when stored at 20°C for 7 days (Ferreira and Lund, 1996). When nisin was added 2000 IU/g to the cottage cheese, there was rise in the inactivation rate up to 3-log10 cycles within 3 days. It was also observed that nisin when added to ricotta-type cheese at a temperature of 6–8°C for a period of 70 days was effective in controlling the growth of *L. monocytogenes* (Davies et al., 1997). Depending on the type of cheese, the addition of 1000 IU ml of nisin can inhibit the growth of *L. monocytogenes* effectively over a period of 8 weeks whereas the control cheese was contaminated after 1–2 weeks with a high level of unwanted organisms. Nisin-producing *lactococcus* produces cheddar cheese containing nisin which is used in the pasteurized processed cheese or cold stored cheese spreads (Zottola et al., 1994). It was further observed that the shelf life increased greatly in pasteurized processed cheese containing nisin, when compared to control cheese spreads. Nisin when added in 100 and 300 IU g to cold packed cheese spreads reduce the growth of *Staphylococcus aureus, L. monocytogenes* and also spores of *C. sporogenes*. One problem associated with the production of cheese is *Clostridium*-related butyric acid fermentation. This problem can be overcome by the addition of nisin to pasteurized cheese spread as it inhibits the growth spores of clostridia like *Clostridium ttyrobutyricum* (Schillinger et al., 1996)*.*

Lacticin 3147 is bacteriocin produced by *Lactobacillus lactis* and is usually broad spectrum, and is a 2-component bacteriocin. It is mainly utilized in maintaining cheddar cheese quality by decreasing the number of non-starter LAB during ripening (Ross et al., 1999). The transconjugant of lacticin 3147 is also used as a protective culture for preventing the growth of *Listeria* on the mold-ripened cheese surface. There is a reduction in the number of *L. monocytogenes* 3-log10 cycles when lacticin 3147 is added on the cheese surface (Ross et al., 1999).

### Probiotics

The gastrointestinal tracts of humans contain a combination of intestinal microbes and the host which coexist. For the development of the mucosal immune system, the gastrointestinal microflora acts as a stimulus (Deplancke and Gaskins, 2002). Two classes of LAB influence the gastrointestinal microflora which mainly consists of 56 types of *Lactobacillus spp.* and various *Bifidobacterium spp.;* these species have shown bacteriocins productions which are *in vitro* in nature (Avonts and De Vuyst, 2001; Cross, 2002). However, recent studies have shown few of these strains producing bacteriocins *in vivo*, one of which is *Lactobacillus salivarius UCC118* that produces bacteriocin in a broad spectrum that is effective against the food-borne pathogen *Listeria monocytogenes* (Claesson et al., 2006).

### Bacteriocin immunity

Bacteriocin can be differentiated from antibiotics by the cell immunity synthesizing bacteriocin to its products. Moreover, the immunity proteins are coded by genes which show a closeness in gene proximity to other bacteriocins of structural and processing genes (Siegers and Entian, 1995). It is normal for the primary bacteriocin gene and the immunity gene to be situated on a similar operon and frequently next to one another (Klein et al., 1993; Nes et al., 1996).

Earlier, it was considered that the immunity of lantibiotics was because of an immunity gene, like nisI for nisin and spaI for subtilin, which code for NisI, SpaI immunity proteins. But in reality, the bacteriocin immunity is the consequence of the impact of several proteins as the deletion of the genes brings an alteration in the immunity of the host (Klein et al., 1993). For instance, non-nisin delivering strains of *Lactobacillus lactis* which are nisin resistant do not have the NisI protein, but they have similar types of sequences to nisF, nisE, and nisG (Duan et al., 1996). The identification of two lantibiotic immunity systems in the producing cells have been carried out. Protection can be interceded by immunity proteins, LanI, and ABC-transport proteins, LanFEG, which can be encoded on various open-understanding frames (Reis et al., 1994; Siegers and Entian, 1995).

The protection of the producing cells obtained from their own bacteriocins was carried out by the synergistic working of these two immunity systems (Klein and Entian, 1994). LanI has the ability to give producer cells immunity, by the prevention of pore formation by the bacteriocin, and this lanI is present on the outer membrane of the cytoplasm. LanFEG evidently acts by transporting bacteriocin atoms that have embedded into the membrane back to the encompassing medium, thus maintaining the concentration of the bacteriocin in the membrane under a critical level.

The non-lantibiotics (Class II bacteriocins) have immunity which are simpler than those of the lantibiotics. For class II bacteriocins, the immunity proteins code for a committed protein that is loosely connected with the cytoplasmic membrane. Western blot (immunoblot) examination showed that the significant part of the immunity protein CbiB2 of carnobacteriocin B2 is tracked down in the cytoplasm; furthermore, that a very smaller portion is related to the membrane. It is seen that the greater part of the immunity protein MesI of mesentericin Y105 is in the cytoplasm, with just a little section recognized in the membrane (Dayem et al., 1996). The immunity protein, which is cationic in nature and whose sizes range between 51 and 254 amino acids, gives complete immunity against the bacteriocin (Nissen-Meyer et al., 1993; Venema et al., 1994).

### Bacteriocin toxicity

LAB produces bacteriocins which have been consumed for a long time. Nisin is approved by the Food and Drug Administration and has been proven safe for regular human consumption at a measured quantity of 2.9 mg/person/day by intense, subchronic, and chronic toxicity studies. Further studies including reproduction, cross resistance, sensitization have proved that nisin is harmless *in vitro* (Frazer et al., 1962; Cleveland et al., 2002).

After checking the effect on pigs and rats, a well-performed proposal was put together that nisin is safe as a preservative. Since nisin is consumed orally, the impact of nisin on the oral microflora was also analyzed. Analysis was carried out 1 min after the consumption of nisin-containing chocolate milk. It was observed that only 1r40 of the activity of the original nisin could be detected in the saliva, compared to the 1r100 activity of the control saliva. Interestingly, a similar study showed penicillin-containing chocolate milk provided the saliva more antibacterial action for a more specific time span (Claypool et al., 1966). Another review showed that gastric enzymes have some impact on nisin. Trypsin inactivated the peptide, and it was inferred that ingested nisin would not have an effect on the microflora of the stomach (Vaucher et al., 2011).

Almost certainly, more information regarding the safety of nisin is not accessible to people in general. New data or information are not being used by patents that claim that nisin is safe and has antibacterial property so can be used in for food and medical applications (Blackburn et al., 1989). The patents does not even check the complete toxicological data before submitting patents on new bacteriocins. However, nisin is at present the most commercially utilized bacteriocin. The safety of other bacteriocins is yet to be investigated for their application in the food and medical industries. Pediocin PA-1 ŽAcH was infused into mice and rabbits, and immunoblotting showed that it was non-immunogenic in both of them (Bhunia et al., 1990).

## Regulatory consideration of using bacteriocins

In some countries, the discrimination between antibiotics and bacteriocins is found to be critical from a regulatory perspective. According to the FDA, the usage of antibiotics in food is strictly prohibited, for example, specific bacteria have been used in Denmark in order to produce food additives which must not produce any antibiotics or toxins (Klaenhammer, 1988). In the United States, the microorganisms which is Generally Recognized as Safe (GRAS) and have been used in the food industry since 1958 are considered as bacteriocin-producing starter cultures (Muriana, 1996). According to the Code of Federal Regulations, the purified bacteriocin which is used as a food preservative by any company should be proclaimed as GRAS, although the rationalization of this self-proclamation is required by the Food and Drug Administration (FDA). “E” numbers are provided to all the food additives by the European Union. For example, nisin is registered as E234, also noted as “nisin preservatives” or “natural preservatives”. In 1988, nisin had obtained the confirmation as Generally Recognized as Safe in the United States by the FDA. The USDA had published guidelines for the approval of new bacteriocins in 1993, which stated that chemically identified and characterized bacteriocins with their efficacy are approved for commercial uses. The approval also needs some documentation regarding manufacture process, quantification, and standardization assays, with toxicological data and the fate of the molecule after consumption.

## Resistance mechanism of bacteriocin

When a new preservative is found to be safe, its longevity of utilization is checked by preventing resistant cell proliferation. Already, cells show resistance to many antibiotics, and therefore the transferal of resistance between organisms has been recorded. Though bacteriocins are different from antibiotics, it is feared that bacteriocin will transform cells into an antibiotic-resistant type. Nisin is seen to not affect the resistance frequency of *L. monocytogenes* Scott to ampicillin and chloramphenicol as both the antibiotics and nisin have totally different modes of activity (Crandall and Montville, 1998). A study showed that various multi-drug resistant microorganisms were treated with 400 lUrml nisin, and the organisms showed sensitivity nisin (Severina et al., 1998). A study showed the nisin was cross-resistant with 33 alternative antimicrobials and antibiotic resistant *Staphylococcus aureus* (Szybalski and Bryson, 1952). Not only bacteriocins but also some cationic peptides also show effectivity against antibiotic resistant strains, like methicillin resistant *Staphylococcus aureus*, and vancomycin-safe *Staphylococcus haemolyticus* (Friedrich et al., 2000). However nisin-resistant microbes do not show antibiotic cross-resistance. But the actual mechanism of resistance is important when studying to avoid the phenomenon. Antibiotic resistance can be attributed to genetic factors that help in the transferring of resistance to cells, strains, and species. In contrast to most antibiotic resistance, bacteriocin opposition results from a physiological change in the objective cell membrane (Ming and Daeschel, 1993; Mazzotta et al., 1997; Crandall and Montville, 1998).

For *L. monocytogenes*, an increase in tolerance to nisin was due to lower C15:C17 ratio (Mazzotta et al., 1997). It was also found that nisin-resistant *L. monocytogenes* decreased levels of phosphatidylglycerol, di phosphatidylglycerol, and bis phosphatidylglycerol phosphate (Ming and Daeschel, 1993). However, most research showed that an alteration of cell membrane composition results in mutants producing enzymes, resistance and also nisinase, an enzyme-degrading nisin (Jarvis, 1967; Gravesen et al., 2000).

It was further recorded that resistant *L. monocytogenes* to pediocin PA-1 showed enhanced expression of gene encoding the b-glucoside-particular phosphoenolpyruvate-dependent phosphotransferase systems. The mechanism by which b-glucoside-explicit PTS cooperates with pediocin to cause resistance should be explained. In research on the mode of activity of mesentericin Y105, a bacteriocin bactericidal against *L. monocytogenes*, transposon mutants resistant to the bacteriocin came about because of the transposon inclusion into a gene ŽrpoN. encoding a putative s 54 factors (Robichon et al., 1997). Whether resistance is genetically encoded or the consequence of a transformation, there is problematic information with respect to cross-resistance when bacteriocins from various classes are utilized (Mazzotta et al., 1997; Crandall and Montville, 1998; Rasch and Knøchel, 1998).

## Genetically engineered bacteriocin

The growing popularity of development of bacteriocins that are not affected by heat processes involved in the food matrix containing the enzymes and the increased solubility and food system distribution is very important for the bacteriocins being successfully used as food preservatives. It caused genetic alteration of the naturally accessible bacteriocins to give out useful physico-chemical properties upgrading its activity in the food framework. The genetic change of nisin A to nisin Z, which has amino acid substitute in His31/Asn27 position, has improved its dispersion in the food lattice seven-fold, contrasted with its natural partner (Mulders et al., 1991; Ross et al., 1993; Cotter et al., 2005).

Class II bacteriocins have linear peptides which are simple targets for genetic alteration, since they go through lesser post translational change and can be heterologously expressed in other nonfastidious hosts, which can be utilized in various processes as they act as good starter cultures. Nisin is the most broadly studied class I bacteriocins, bacteriocin for genetic alterations, which consists of thiol bridge mutations, alterations made to the composition of uncommon amino acid of the peptide, changes altering the pivot region of the peptide and changes to the total charge of the peptide (Ross et al., 1993; Cotter et al., 2005). Mutations including amino acids like dehydrobutyrine (Dhb) in the variations Dhb14S and A12L presented critical resistance from trypsin, yet undermined its antimicrobial activity. Increased hydrophobicity of the variant of subtilisin by altering its N-terminal area is accomplished by genetic changes, which upgraded its action threefold more than natural subtilisin (Liu and Hansen, 1992).

The solubility and antimicrobial activity is increased against gram-negative food pathogenic microbes like *Shigella* spp., *Pseudomonas spp*. due to mutations of the nisin’s hinge part (Chen et al., 1998). It is also observed that bacteriocin solubility is also increased by mutation within the same region even at higher pH like pH 8, and also increased heat stability at neutral pH (Yuan et al., 2004).

Microcin J25 is modified to become suitable for enzymatic digestion with the help of chymotrypsin, maintaining the safety of the bacteriocin so that it is still suitable for human consumption (Pomares et al., 2009). The disulfide bridge that is present in the C-terminus domain of the peptide of some bacteriocins is responsible for increasing antimicrobial activity at higher temperatures (Fimland et al., 2000). Class II bacteriocins like pediocin are not stable at room temperature or low storage temperature conditions, but this problem can be solved by using residues of methionine in the bacteriocin to hydrophobic residues as a result of which there is increased peptide stability, and the longevity of the antimicrobial activity is also increased (Crameri et al., 1998).

The bacteriocins having genetic modifications are first evaluated through strict safety regulatory tests and various norms which are approved by the FDA, after which they are allowed to be used for human consumption. LAB has the self-cloning property which includes alterations of the plasmid with the help of gene knockout, overlap extension splicing, and site directed mutagenesis (Ross et al., 1993; Cotter et al., 2005).

The genetically engineered bacteriocins have to follow the guidelines approved by the FDA for their usage, which are as follows:

I. The safety of the genetic material that is to be used should be confirmed. Preference is to be given to the genetic material or DNA obtained from organisms that are being used already in food systems. The total genetic material utilized in the artificial constructs should be confirmed and characterized. It should be evaluated that no extra genetic material present, and both the donor and the host should be characterized.
II. The host organism’s identification and origin should be well-featured, and also its safety should be confirmed. There should be further testing on the presence of factors like virulence, toxins etc. The vectors utilized in such genetic constructions should have been obtained from organisms identified as secure for usage in food. The selectable marker found in such vectors should now no longer encode resistance to antibiotics utilized in therapeutic interventions.
III. Some harmful traits like pathogenicity, toxigenicity, and allergenicity should be totally removed, and verification should be carried out by studies on both *in vitro* and animals. There should be complete absence of pleitrophic effects with respect to chemicals, and the organoleptic property of the food as compared to the non-engineered types.
IV. Evaluation of the nutrient composition of food is carried out where genetically engineered starter cultures are utilized. Further evaluation of the exposure levels of bacteriocins of them in the consumer population are carried out.

The regulations for assessing genetically engineered bacteriocins are not fixed, and the organisms are analyzed solely on their usage of the products. LAB strains were genetically modified for increasing the production of bacteriocins (ChenHoover, 2003). The non-natural producers of bacteriocins which are basically heterologous bactericon expression of the host are recognized nowadays, due to the following reasons:

1. The naturally producing strains of LAB require special conditions and nutrient composition for their optimal growth. It further enhances bacteriocin expression in the host which has minimalistic media requirements, and hence there is a reduction in the production as well as purification cost and production of bacteriocin to a large extent.
2. It is also seen that some of the bacteriocin-producing strains have harmful effects on the food processes that mainly utilize starter cultures for their fermentation processes.
3. All the producing strains are not capable of being effective in all food matrices and produce the desired amount of bacteriocins, and they are also not effective enough to give protection to foods.
4. The harmful and toxic characteristics of the producing strain and the strain having antimicrobial resistance makes them not suitable to be utilized in the food systems.

## References

[B1] Abee T. Krockel L. Hill C. (1995). Bacteriocins: Modes of action and potentials in food preservation and control of food poisoning. Int. J. Food Microbiol. 28, 169–185. 10.1016/0168-1605(95)00055-0 8750665

[B2] Altena K. Guder A. Cramer C. Bierbaum G. (2000). Biosynthesis of the lantibiotic mersacidin: Organization of a type B lantibiotic gene cluster. Appl. Environ. Microbiol. 66, 2565–2571. 10.1128/AEM.66.6.2565-2571.2000 10831439 PMC110582

[B3] Alvarez-Sieiro P. Montalbán-López M. Mu D. Kuipers O. P. (2016). Bacteriocins of lactic acid bacteria: Extending the family. Appl. Microbiol. Biotechnol. 100, 2939–2951. 10.1007/s00253-016-7343-9 26860942 PMC4786598

[B4] Ariyapitipun T. Mustapha A. Clarke A. D. (1999). Microbial shelf life determination of vacuum-packaged fresh beef treated with polylactic acid, lactic acid, and nisin solutions. J. Food Prot. 62, 913–920. 10.4315/0362-028x-62.8.913 10456746

[B5] Ariyapitipun T. Mustapha A. Clarke A. D. (2000). Survival of Listeria monocytogenes Scott A on vacuum-packaged raw beef treated with polylactic acid, lactic acid, and nisin. J. Food Prot. 63, 131–136. 10.4315/0362-028x-63.1.131 10643784

[B6] Avonts L. De Vuyst L. (2001). Antimicrobial potential of probiotic lactic acid bacteria. Meded. Rijksuniv. Gent. Fak. Landbouwkd. Toegep. Biol. Wet. 66, 543–550. 15954651

[B7] Aymerich T. Artigas M. G. Garriga M. Monfort J. M. Hugas M. (2000). Effect of sausage ingredients and additives on the production of enterocin A and B by Enterococcus faecium CTC492. Optimization of *in vitro* production and anti-listerial effect in dry fermented sausages. J. Appl. Microbiol. 88, 686–694. 10.1046/j.1365-2672.2000.01012.x 10792528

[B8] Baccus-Taylor G. Glass K. A. Luchansky J. B. Maurer A. J. (1993). Fate of Listeria monocytogenes and pediococcal starter cultures during the manufacture of chicken summer sausage. Poult. Sci. 72, 1772–1778. 10.3382/ps.0721772 8234138

[B9] Balciunas E. M. Castillo Martinez F. A. Todorov S. D. Franco B. D. G. de M. Converti A. Oliveira R. P. de S. (2013). Novel biotechnological applications of bacteriocins: A review. Food control. 32, 134–142. 10.1016/j.foodcont.2012.11.025

[B10] Berry E. D. Hutkins R. W. Mandigo R. W. (1991). The use of bacteriocin-producing Pediococcus acidilactici to control postprocessing Listeria monocytogenes contamination of frankfurters (1). J. Food Prot. 54, 681–686. 10.4315/0362-028X-54.9.681 31051573

[B11] Bhunia A. K. Johnson M. C. Ray B. Belden E. L. (1990). Antigenic property of pediocin AcH produced by Pediococcus acidilactici H. J. Appl. Bacteriol. 69, 211–215. 10.1111/j.1365-2672.1990.tb01511.x 2272942

[B12] Bierbaum G. Sahl H.-G. (2009). Lantibiotics: Mode of action, biosynthesis and bioengineering. Curr. Pharm. Biotechnol. 10, 2–18. 10.2174/138920109787048616 19149587

[B13] Blackburn P. Polak J. Gusik S. Rubino S, D. (1989). Nisin compositions for use as enhanced. broad range bactericides 1–40. US Patent no: US5217950A.

[B14] Bo L. Daniel S. Libusha K. Yanxiang S. Katherine H. Ike J. (2010). Catalytic promiscuity in the biosynthesis of cyclic peptide secondary metabolites in planktonic marine cyanobacteria. Proc. Natl. Acad. Sci. U. S. A. 107, 10430–10435. 10.1073/pnas.0913677107 20479271 PMC2890784

[B15] Bruno M. E. Kaiser A. Montville T. J. (1992). Depletion of proton motive force by nisin in Listeria monocytogenes cells. Appl. Environ. Microbiol. 58, 2255–2259. 10.1128/aem.58.7.2255-2259.1992 1637163 PMC195764

[B16] Campanini M. Pedrazzoni I. Barbuti S. Baldini P. (1993). Behaviour of Listeria monocytogenes during the maturation of naturally and artificially contaminated salami: Effect of lactic-acid bacteria starter cultures. Int. J. Food Microbiol. 20, 169–175. 10.1016/0168-1605(93)90109-t 8312141

[B17] Cascales E. Buchanan S. K. Duché D. Kleanthous C. Lloubès R. Postle K. (2007). Colicin biology. Microbiol. Mol. Biol. Rev. 71, 158–229. 10.1128/MMBR.00036-06 17347522 PMC1847374

[B18] Cebrián R. Martínez-Bueno M. Valdivia E. Albert A. Maqueda M. Sánchez-Barrena M. J. (2015). The bacteriocin AS-48 requires dimer dissociation followed by hydrophobic interactions with the membrane for antibacterial activity. J. Struct. Biol. X. 190, 162–172. 10.1016/j.jsb.2015.03.006 25816760

[B19] Chatterjee C. Paul M. Xie L. van der Donk W. A. (2005). Biosynthesis and mode of action of lantibiotics. Chem. Rev. 105, 633–684. 10.1021/cr030105v 15700960

[B20] Chen P. Novak J. Kirk M. Barnes S. Qi F. Caufield P. W. (1998). Structure-activity study of the lantibiotic mutacin II from Streptococcus mutans T8 by a gene replacement strategy. Appl. Environ. Microbiol. 64, 2335–2340. 10.1128/AEM.64.7.2335-2340.1998 9647795 PMC106391

[B21] Chung K. T. Dickson J. S. Crouse J. D. (1989). Effects of nisin on growth of bacteria attached to meat. Appl. Environ. Microbiol. 55, 1329–1333. 10.1128/aem.55.6.1329-1333.1989 2764559 PMC202866

[B22] Cintas L. M. Casaus M. P. Herranz C. Nes I. F. Hernández P. E. (2001). Review: Bacteriocins of lactic acid bacteria. Food Sci. Technol. Int. 7, 281–305. 10.1106/R8DE-P6HU-CLXP-5RYT

[B23] Claesson M. J. Li Y. Leahy S. Canchaya C. van Pijkeren J. P. Cerdeño-Tárraga A. M. (2006). Multireplicon genome architecture of Lactobacillus salivarius. Proc. Natl. Acad. Sci. U. S. A. 103, 6718–6723. 10.1073/pnas.0511060103 16617113 PMC1436024

[B24] Claypool L. Heinemann B. Voris L. Stumbo C. R. (1966). Residence time of nisin in the oral cavity following consumption of chocolate milk containing nisin. J. Dairy Sci. 49, 314–316. 10.3168/jds.S0022-0302(66)87855-4 6012851

[B25] Cleveland J. Chikindas M. Montville T. J. (2002). Multimethod assessment of commercial nisin preparations. J. Ind. Microbiol. Biotechnol. 29, 228–232. 10.1038/sj.jim.7000315 12407455

[B26] Cleveland J. Montville T. J. Nes I. F. Chikindas M. L. (2001). Bacteriocins: Safe, natural antimicrobials for food preservation. Int. J. Food Microbiol. 71, 1–20. 10.1016/s0168-1605(01)00560-8 11764886

[B27] Cotter P. D. Hill C. Ross R. P. (2005). Bacteriocins: Developing innate immunity for food. Nat. Rev. Microbiol. 3, 777–788. 10.1038/nrmicro1273 16205711

[B28] Cox C. R. Coburn P. S. Gilmore M. S. (2005). Enterococcal cytolysin: A novel two component peptide system that serves as a bacterial defense against eukaryotic and prokaryotic cells. Curr. Protein Pept. Sci. 6, 77–84. 10.2174/1389203053027557 15638770

[B29] Crameri A. Raillard S. A. Bermudez E. Stemmer W. P. (1998). DNA shuffling of a family of genes from diverse species accelerates directed evolution. Nature 391, 288–291. 10.1038/34663 9440693

[B30] Crandall A. D. Montville T. J. (1998). Nisin resistance in Listeria monocytogenes ATCC 700302 is a complex phenotype. Appl. Environ. Microbiol. 64, 231–237. 10.1128/AEM.64.1.231-237.1998 9435079 PMC124699

[B31] Cross M. L. (2002). Microbes versus microbes: Immune signals generated by probiotic lactobacilli and their role in protection against microbial pathogens. FEMS Immunol. Med. Microbiol. 34, 245–253. 10.1111/j.1574-695X.2002.tb00632.x 12443824

[B32] Cutter C. N. Siragusa G. R. (1998). Incorporation of nisin into a meat binding system to inhibit bacteria on beef surfaces. Lett. Appl. Microbiol. 27, 19–23. 10.1046/j.1472-765x.1998.00381.x 9722992

[B33] Davies E. A. Bevis H. E. Delves-Broughton J. (1997). The use of the bacteriocin, nisin, as a preservative in ricotta-type cheeses to control the food-borne pathogen Listeria monocytogenes. Lett. Appl. Microbiol. 24, 343–346. 10.1046/j.1472-765x.1997.00145.x 9172439

[B34] Davies E. A. Milne C. F. Bevis H. E. Potter R. W. Harris J. M. Williams G. C. (1999). Effective use of nisin to control lactic acid bacterial spoilage in vacuum-packed bologna-type sausage. J. Food Prot. 62, 1004–1010. 10.4315/0362-028x-62.9.1004 10492474

[B35] Dayem M. A. Fleury Y. Devilliers G. Chaboisseau E. Girard R. Nicolas P. (1996). The putative immunity protein of the gram-positive bacteria Leuconostoc mesenteroides is preferentially located in the cytoplasm compartment. FEMS Microbiol. Lett. 138, 251–259. 10.1111/j.1574-6968.1996.tb08166.x 9026455

[B36] Deegan L. H. Cotter P. D. Hill C. Ross P. (2006). Bacteriocins: Biological tools for bio-preservation and shelf-life extension. Int. Dairy J. 16, 1058–1071. 10.1016/j.idairyj.2005.10.026

[B37] Degnan A. J. Yousef A. E. Luchansky J. B. (1992). Use of Pediococcus acidilactici to control Listeria monocytogenes in temperature-abused vacuum-packaged wieners. J. Food Prot. 55, 98–103. 10.4315/0362-028X-55.2.98 31071771

[B38] Delves-Broughton J. Blackburn P. Evans R. J. Hugenholtz J. (1996). Applications of the bacteriocin, nisin. Ant. Van Leeuwenhoek 69, 193–202. 10.1007/BF00399424 8775979

[B39] Deplancke B. Gaskins H. R. (2002). Redox control of the transsulfuration and glutathione biosynthesis pathways. Curr. Opin. Clin. Nutr. Metab. Care 5, 85–92. 10.1097/00075197-200201000-00015 11790955

[B40] Diep D. B. Nes I. F. (2002). Ribosomally synthesized antibacterial peptides in Gram positive bacteria. Curr. Drug Targets 3, 107–122. 10.2174/1389450024605409 11958295

[B41] Dirix G. Monsieurs P. Dombrecht B. Daniels R. Marchal K. Vanderleyden J. (2004). Peptide signal molecules and bacteriocins in gram-negative bacteria: A genome-wide *in silico* screening for peptides containing a double-glycine leader sequence and their cognate transporters. Peptides 25, 1425–1440. 10.1016/j.peptides.2003.10.028 15374646

[B42] Drider D. Fimland G. Héchard Y. McMullen L. M. Prévost H. (2006). The continuing story of class IIa bacteriocins. Microbiol. Mol. Biol. Rev. 70, 564–582. 10.1128/MMBR.00016-05 16760314 PMC1489543

[B43] Duan K. Harvey M. L. Liu C. Q. Dunn N. W. (1996). Identification and characterization of a mobilizing plasmid, pND300, in Lactococcus lactis M189 and its encoded nisin resistance determinant. J. Appl. Bacteriol. 81, 493–500. 10.1111/j.1365-2672.1996.tb03538.x 8939027

[B44] Dykes G. A. (1995). Bacteriocins: Ecological and evolutionary significance. Trends Ecol. Evol. 10, 186–189. 10.1016/S0169-5347(00)89049-7 21236999

[B45] Einarsson H. Lauzon H. L. (1995). Biopreservation of brined shrimp (pandalus borealis) by bacteriocins from lactic acid bacteria. Appl. Environ. Microbiol. 61, 669–676. 10.1128/aem.61.2.669-676.1995 16534936 PMC1388354

[B46] El Kafsi H. Loux V. Mariadassou M. Blin C. Chiapello H. Abraham A.-L. (2017). Unprecedented large inverted repeats at the replication terminus of circular bacterial chromosomes suggest a novel mode of chromosome rescue. Sci. Rep. 7, 44331. 10.1038/srep44331 28281695 PMC5345009

[B47] Engelke G. Gutowski-Eckel Z. Kiesau P. Siegers K. Hammelmann M. Entian K. D. (1994). Regulation of nisin biosynthesis and immunity in Lactococcus lactis 6F3. Appl. Environ. Microbiol. 60, 814–825. 10.1128/aem.60.3.814-825.1994 8161176 PMC201397

[B48] Ennahar S. Aoude-Werner D. Sorokine O. Van Dorsselaer A. Bringel F. Hubert J. C. (1996). Production of pediocin AcH by Lactobacillus plantarum WHE 92 isolated from cheese. Appl. Environ. Microbiol. 62, 4381–4387. 10.1128/aem.62.12.4381-4387.1996 8953710 PMC168265

[B49] Eppert I. Valdés-Stauber N. Götz H. Busse M. Scherer S. (1997). Growth reduction of Listeria spp. caused by undefined industrial red smear cheese cultures and bacteriocin-producing Brevibacterium lines as evaluated *in situ* on soft cheese. Appl. Environ. Microbiol. 63, 4812–4817. 10.1128/aem.63.12.4812-4817.1997 9406400 PMC168805

[B50] Ferreira M. A. Lund B. M. (1996). The effect of nisin on Listeria monocytogenes in culture medium and long-life cottage cheese. Lett. Appl. Microbiol. 22, 433–438. 10.1111/j.1472-765x.1996.tb01197.x 8695069

[B51] Fimland G. Johnsen L. Axelsson L. Brurberg M. B. Nes I. F. Eijsink V. G. (2000). A C-terminal disulfide bridge in pediocin-like bacteriocins renders bacteriocin activity less temperature dependent and is a major determinant of the antimicrobial spectrum. J. Bacteriol. 182, 2643–2648. 10.1128/JB.182.9.2643-2648.2000 10762272 PMC111334

[B52] Fimland G. Johnsen L. Dalhus B. Nissen-Meyer J. (2005). Pediocin-like antimicrobial peptides (class IIa bacteriocins) and their immunity proteins: Biosynthesis, structure, and mode of action. J. Pept. Sci. 11, 688–696. 10.1002/psc.699 16059970

[B53] Foegeding P. M. Thomas A. B. Pilkington D. H. Klaenhammer T. R. (1992). Enhanced control of Listeria monocytogenes by *in situ*-produced pediocin during dry fermented sausage production. Appl. Environ. Microbiol. 58, 884–890. 10.1128/aem.58.3.884-890.1992 1575489 PMC195349

[B54] Franke C. M. Leenhouts K. J. Haandrikman A. J. Kok J. Venema G. Venema K. (1996). Topology of LcnD, a protein implicated in the transport of bacteriocins from Lactococcus lactis. J. Bacteriol. 178, 1766–1769. 10.1128/jb.178.6.1766-1769.1996 8626308 PMC177865

[B55] Frazer A. C. Sharratt M. Hickman J. R. (1962). The biological effects of food additives. I.—Nisin. J. Sci. Food Agric. 13, 32–42. 10.1002/jsfa.2740130106

[B56] Friedrich C. L. Moyles D. Beveridge T. J. Hancock R. E. (2000). Antibacterial action of structurally diverse cationic peptides on gram-positive bacteria. Antimicrob. Agents Chemother. 44, 2086–2092. 10.1128/AAC.44.8.2086-2092.2000 10898680 PMC90018

[B57] Gebhard S. (2012). ABC transporters of antimicrobial peptides in Firmicutes bacteria - phylogeny, function and regulation. Mol. Microbiol. 86, 1295–1317. 10.1111/mmi.12078 23106164

[B58] Gravesen A. Warthoe P. Knøchel S. Thirstrup K. (2000). Restriction fragment differential display of pediocin-resistant Listeria monocytogenes 412 mutants shows consistent overexpression of a putative β-glucoside-specific PTS system the EMBL accession numbers for the sequences reported in this paper are AJ251202, AJ251203 and AJ251204. Microbiology 146 (6), 1381–1389. 10.1099/00221287-146-6-1381 10846216

[B59] Chen H. Hoover D. G. (2003). Bacteriocins and their food applications. Compr. Rev. food Sci. food Saf. 2, 82–100. 10.1111/j.1541-4337.2003.tb00016.x 33451234

[B60] Haddad Kashani H. Nikzad H. Mobaseri S. Hoseini E. (2012). Synergism effect of nisin peptide in reducing chemical preservatives in food industry. Life Sci. J. 9, 496–501.

[B61] Håvarstein L. S. Holo H. Nes I. F. (1994). The leader peptide of colicin V shares consensus sequences with leader peptides that are common among peptide bacteriocins produced by gram-positive bacteria. Microbiology 140 (9), 2383–2389. 10.1099/13500872-140-9-2383 7952189

[B62] Héchard Y. Sahl H. G. (2002). Mode of action of modified and unmodified bacteriocins from Gram-positive bacteria. Biochimie 84, 545–557. 10.1016/s0300-9084(02)01417-7 12423799

[B63] Horn N. Martínez M. I. Martínez J. M. Hernández P. E. Gasson M. J. Rodríguez J. M. (1999). Enhanced production of pediocin PA-1 and coproduction of nisin and pediocin PA-1 by Lactococcus lactis. Appl. Environ. Microbiol. 65, 4443–4450. 10.1128/AEM.65.10.4443-4450.1999 10508073 PMC91591

[B64] Jack R. W. Wan J. Gordon J. Harmark K. Davidson B. E. Hillier A. J. (1996). Characterization of the chemical and antimicrobial properties of piscicolin 126, a bacteriocin produced by Carnobacterium piscicola JG126. Appl. Environ. Microbiol. 62, 2897–2903. 10.1128/aem.62.8.2897-2903.1996 8702282 PMC168075

[B65] Jarvis B. (1967). Resistance to nisin and production of nisin-inactivating enzymes by several Bacillus species. J. Gen. Microbiol. 47, 33–48. 10.1099/00221287-47-1-33 4962191

[B66] Jay J. M. Loessner M. J. Golden D. A. (Editors) (2005). Other food protection methods BT - modern food microbiology (Boston, MA: Springer US), 457–470. 978-0-387-23413-7.

[B67] Johnson E. M. Jung D. Y.-G. Jin D. Y.-Y. Jayabalan D. R. Yang D. S. H. Suh J. W. (2018). Bacteriocins as food preservatives: Challenges and emerging horizons. Crit. Rev. Food Sci. Nutr. 58, 2743–2767. 10.1080/10408398.2017.1340870 28880573

[B68] Kankainen M. Paulin L. Tynkkynen S. von Ossowski I. Reunanen J. Partanen P. (2009). Comparative genomic analysis of Lactobacillus rhamnosus GG reveals pili containing a human- mucus binding protein. Proc. Natl. Acad. Sci. U. S. A. 106, 17193–17198. 10.1073/pnas.0908876106 19805152 PMC2746127

[B69] Katla T. Møretrø T. Aasen I. M. Holck A. Axelsson L. Naterstad K. (2001). Inhibition of Listeria monocytogenes in cold smoked salmon by addition of sakacin P and/or liveLactobacillus sakei cultures. Food Microbiol. 18, 431–439. 10.1006/fmic.2001.0420

[B70] Kierończyk B. Rawski M. Mikołajczak Z. Świątkiewicz S. Józefiak D. (2020). Nisin as a novel feed additive: The effects on gut microbial modulation and activity, histological parameters, and growth performance of broiler chickens. Anim. (Basel). 10, 101. 10.3390/ani10010101 PMC7023484 31936255

[B71] Kirtonia K. Salauddin M. Bharadwaj K. K. Pati S. Dey A. Shariati M. A. (2021). Bacteriocin: A new strategic antibiofilm agent in food industries. Biocatal. Agric. Biotechnol. 2021, 102141. 10.1016/j.bcab.2021.102141

[B72] Klaenhammer T. R. (1988). Bacteriocins of lactic acid bacteria. Biochimie 70, 337–349. 10.1016/0300-9084(88)90206-4 3139051

[B73] Klaenhammer T. R. (1993). Genetics of bacteriocins produced by lactic acid bacteria. FEMS Microbiol. Rev. 12, 39–85. 10.1016/0168-6445(93)90057-g 8398217

[B74] Kleerebezem M. Quadri L. E. Kuipers O. P. de Vos W. M. (1997). Quorum sensing by peptide pheromones and two-component signal-transduction systems in Gram-positive bacteria. Mol. Microbiol. 24, 895–904. 10.1046/j.1365-2958.1997.4251782.x 9219998

[B75] Klein C. Entian K. D. (1994). Genes involved in self-protection against the lantibiotic subtilin produced by Bacillus subtilis ATCC 6633. Appl. Environ. Microbiol. 60, 2793–2801. 10.1128/aem.60.8.2793-2801.1994 8085823 PMC201725

[B76] Klein C. Kaletta C. Entian K. D. (1993). Biosynthesis of the lantibiotic subtilin is regulated by a histidine kinase/response regulator system. Appl. Environ. Microbiol. 59, 296–303. 10.1128/aem.59.1.296-303.1993 8439156 PMC202094

[B77] Koponen O. Tolonen M. Qiao M. Wahlström G. Helin J. Saris P. E. J. (2002). NisB is required for the dehydration and NisC for the lanthionine formation in the post-translational modification of nisin. Microbiology 148, 3561–3568. 10.1099/00221287-148-11-3561 12427947

[B78] Kotelnikova E. A. GelfandBacteriocin M. S. (2002). Production by gram-positive bacteria and the mechanisms of transcriptional regulation. Russ. J. Genet. 38, 628–641. 10.1023/A:1016035700012

[B79] Lahti E. Johansson T. Honkanen-Buzalski T. Hill P. Nurmi E. (2001). Survival and detection of *Escherichia coli* O157:H7 and Listeria monocytogenes during the manufacture of dry sausage using two different starter cultures. Food Microbiol. 18, 75–85. 10.1006/fmic.2000.0373

[B80] Lawton E. M. Ross R. P. Hill C. Cotter P. D. (2007). Two-peptide lantibiotics: A medical perspective. Mini Rev. Med. Chem. 7, 1236–1247. 10.2174/138955707782795638 18220976

[B81] Lee S. W. Mitchell D. A. Markley A. L. Hensler M. E. Gonzalez D. Wohlrab A. (2008). Discovery of a widely distributed toxin biosynthetic gene cluster. Proc. Natl. Acad. Sci. U. S. A. 105, 5879–5884. 10.1073/pnas.0801338105 18375757 PMC2311365

[B82] Leisner J. J. Greer G. G. Stiles M. E. (1996). Control of beef spoilage by a sulfide-producing Lactobacillus sake strain with bacteriocinogenic Leuconostoc gelidum UAL187 during anaerobic storage at 2 degrees C. Appl. Environ. Microbiol. 62, 2610–2614. 10.1128/aem.62.7.2610-2614.1996 8779597 PMC168040

[B83] Leroy F. De Vuyst L. (2004). Lactic acid bacteria as functional starter cultures for the food fermentation industry. Trends Food Sci. Technol. 15, 67–78. 10.1016/j.tifs.2003.09.004

[B84] Linnan M. J. Mascola L. Lou X. D. Goulet V. May S. Salminen C. (1988). Epidemic listeriosis associated with Mexican-style cheese. N. Engl. J. Med. 319, 823–828. 10.1056/NEJM198809293191303 3137471

[B85] Liserre A. M. Landgraf M. Destro M. T. Franco B. D. G. M. (2002). Inhibition of Listeria monocytogenes by a bacteriocinogenic Lactobacillus sake strain in modified atmosphere-packaged Brazilian sausage. Meat Sci. 61, 449–455. 10.1016/s0309-1740(01)00220-0 22061076

[B86] Liu W. Hansen J. N. (1992). Enhancement of the chemical and antimicrobial properties of subtilin by site-directed mutagenesis. J. Biol. Chem. 267, 25078–25085. 10.1016/s0021-9258(19)74008-3 1460009

[B87] Liu W. Hansen J. N. (1990). Some chemical and physical properties of nisin, a small-protein antibiotic produced by Lactococcus lactis. Appl. Environ. Microbiol. 56, 2551–2558. 10.1128/aem.56.8.2551-2558.1990 2119570 PMC184764

[B88] Lu H. Giordano F. Ning Z. (2016). Oxford nanopore MinION sequencing and genome assembly. Genomics Proteomics Bioinforma. 14, 265–279. 10.1016/j.gpb.2016.05.004 PMC5093776 27646134

[B89] Luchansky J. B. Glass K. A. Harsono K. D. Degnan A. J. Faith N. G. Cauvin B. (1992). Genomic analysis of Pediococcus starter cultures used to control Listeria monocytogenes in Turkey summer sausage. Appl. Environ. Microbiol. 58, 3053–3059. 10.1128/aem.58.9.3053-3059.1992 1444419 PMC183047

[B90] Majchrzykiewicz J. A. Lubelski J. Moll G. N. Kuipers A. Bijlsma J. J. E. Kuipers O. P. (2010). Production of a class II two-component lantibiotic of Streptococcus pneumoniae using the class I nisin synthetic machinery and leader sequence. Antimicrob. Agents Chemother. 54, 1498–1505. 10.1128/AAC.00883-09 20100873 PMC2849381

[B91] Makarova K. Slesarev A. Wolf Y. Sorokin A. Mirkin B. Koonin E. (2006). Comparative genomics of the lactic acid bacteria. Proc. Natl. Acad. Sci. U. S. A. 103, 15611–15616. 10.1073/pnas.0607117103 17030793 PMC1622870

[B92] Martin-Visscher L. A. Sprules T. Gursky L. J. Vederas J. C. (2008). Nuclear magnetic resonance solution structure of PisI, a group B immunity protein that provides protection against the type IIa bacteriocin piscicolin 126, PisA. Biochemistry 47, 6427–6436. 10.1021/bi8004076 18500825

[B93] Mazzotta A. S. Crandall A. D. Montville T. J. (1997). Nisin resistance in Clostridium botulinum spores and vegetative cells. Appl. Environ. Microbiol. 63, 2654–2659. 10.1128/aem.63.7.2654-2659.1997 16535641 PMC1389196

[B94] McAuliffe O. Ross R. P. Hill C. (2001). Lantibiotics: Structure, biosynthesis and mode of action. FEMS Microbiol. Rev. 25, 285–308. 10.1111/j.1574-6976.2001.tb00579.x 11348686

[B95] McAuliffe O. Ryan M. P. Ross R. P. Hill C. Breeuwer P. Abee T. (1998). Lacticin 3147, a broad-spectrum bacteriocin which selectively dissipates the membrane potential. Appl. Environ. Microbiol. 64, 439–445. 10.1128/AEM.64.2.439-445.1998 9464377 PMC106063

[B96] McClerren A. L. Cooper L. E. Quan C. Thomas P. M. Kelleher N. L. van der Donk W. A. (2006). Discovery and *in vitro* biosynthesis of haloduracin, a two-component lantibiotic. Proc. Natl. Acad. Sci. U. S. A. 103, 17243–17248. 10.1073/pnas.0606088103 17085596 PMC1859917

[B97] McLaughlin R. E. Ferretti J. J. Hynes W. L. (1999). Nucleotide sequence of the streptococcin A-FF22 lantibiotic regulon: Model for production of the lantibiotic SA-FF22 by strains of Streptococcus pyogenes. FEMS Microbiol. Lett. 175, 171–177. 10.1111/j.1574-6968.1999.tb13616.x 10386366

[B98] Ming X. Daeschel M. A. (1993). Nisin resistance of foodborne bacteria and the specific resistance responses of Listeria monocytogenes Scott A. J. Food Prot. 56, 944–948. 10.4315/0362-028X-56.11.944 31113089

[B99] Mokoena M. P. (2017). Lactic acid bacteria and their bacteriocins: Classification, biosynthesis and applications against uropathogens: A mini-review. Molecules 22, 1255. 10.3390/molecules22081255 PMC6152299 28933759

[B100] Moll G. N. Konings W. N. Driessen A. J. (1999). Bacteriocins: Mechanism of membrane insertion and pore formation. Ant. Van Leeuwenhoek 76, 185–198. 10.1023/a:1002002718501 10532378

[B101] Mulders J. W. Boerrigter I. J. Rollema H. S. Siezen R. J. de Vos W. M. (1991). Identification and characterization of the lantibiotic nisin Z, a natural nisin variant. Eur. J. Biochem. 201, 581–584. 10.1111/j.1432-1033.1991.tb16317.x 1935953

[B102] Müller W. M. Ensle P. Krawczyk B. Süssmuth R. D. (2011). Leader peptide-directed processing of labyrinthopeptin A2 precursor peptide by the modifying enzyme LabKC. Biochemistry 50, 8362–8373. 10.1021/bi200526q 21905643

[B103] Müller W. M. Schmiederer T. Ensle P. Süssmuth R. D. (2010). *In vitro* biosynthesis of the prepeptide of type-III lantibiotic labyrinthopeptin A2 including formation of a C-C bond as a post-translational modification. Angew. Chem. Int. Ed. 49, 2436–2440. 10.1002/anie.200905909 20191635

[B104] Muriana P. M. (1996). Bacteriocins for control of Listeria spp. in food. J. Food Prot. 59, 54–63. 10.4315/0362-028X-59.13.54 28384026

[B105] Murinda S. E. Rashid K. A. Roberts R. F. (2003). *In vitro* assessment of the cytotoxicity of nisin, pediocin, and selected colicins on simian virus 40-transfected human colon and Vero monkey kidney cells with trypan blue staining viability assays. J. Food Prot. 66, 847–853. 10.4315/0362-028x-66.5.847 12747695

[B106] Nes I. F. Diep D. B. Håvarstein L. S. Brurberg M. B. Eijsink V. Holo H. (1996). Biosynthesis of bacteriocins in lactic acid bacteria. Ant. Van Leeuwenhoek 70, 113–128. 10.1007/BF00395929 8879403

[B107] Neville B. A. Forde B. M. Claesson M. J. Darby T. Coghlan A. Nally K. (2012). Characterization of pro-inflammatory flagellin proteins produced by Lactobacillus ruminis and related motile Lactobacilli. PLoS One 7, e40592. 10.1371/journal.pone.0040592 22808200 PMC3393694

[B108] Nielsen J. W. Dickson J. S. Crouse J. D. (1990). Use of a bacteriocin produced by Pediococcus acidilactici to inhibit Listeria monocytogenes associated with fresh meat. Appl. Environ. Microbiol. 56, 2142–2145. 10.1128/aem.56.7.2142-2145.1990 2117881 PMC184573

[B109] Nilsson L. Gram L. Huss H. H. (1999). Growth control of Listeria monocytogenes on cold-smoked salmon using a competitive lactic acid bacteria flora. J. Food Prot. 62, 336–342. 10.4315/0362-028x-62.4.336 10419205

[B110] Nilsson L. Huss H. H. Gram L. (1997). Inhibition of Listeria monocytogenes on cold-smoked salmon by nisin and carbon dioxide atmosphere. Int. J. Food Microbiol. 38, 217–227. 10.1016/s0168-1605(97)00111-6 9506287

[B111] Nissen-Meyer J. Larsen A. G. Sletten K. Daeschel M. Nes I. F. (1993). Purification and characterization of plantaricin A, a Lactobacillus plantarum bacteriocin whose activity depends on the action of two peptides. J. Gen. Microbiol. 139, 1973–1978. 10.1099/00221287-139-9-1973 8245827

[B112] Nuñez M. Rodríguez J. L. García E. Gaya P. Medina M. (1997). Inhibition of Listeria monocytogenes by enterocin 4 during the manufacture and ripening of Manchego cheese. J. Appl. Microbiol. 83, 671–677. 10.1046/j.1365-2672.1997.00275.x 9449804

[B113] Pomares M. F. Salomón R. A. Pavlova O. Severinov K. Farías R. Vincent P. A. (2009). Potential applicability of chymotrypsin-susceptible microcin J25 derivatives to food preservation. Appl. Environ. Microbiol. 75, 5734–5738. 10.1128/AEM.01070-09 19592527 PMC2737906

[B114] Proença J. T. Barral D. C. Gordo I. (2017). Commensal-to-pathogen transition: One-single transposon insertion results in two pathoadaptive traits in *Escherichia coli* -macrophage interaction. Sci. Rep. 7, 4504. 10.1038/s41598-017-04081-1 28674418 PMC5495878

[B115] Rasch M. Knøchel S. (1998). Variations in tolerance of Listeria monocytogenes to nisin, pediocin PA-1 and bavaricin A. Lett. Appl. Microbiol. 27, 275–278. 10.1046/j.1472-765x.1998.t01-6-00449.x 9830144

[B116] Rayman K. Malik N. Hurst A. (1983). Failure of nisin to inhibit outgrowth of Clostridium botulinum in a model cured meat system. Appl. Environ. Microbiol. 46, 1450–1452. 10.1128/aem.46.6.1450-1452.1983 6362566 PMC239594

[B117] Rayman M. K. Aris B. Hurst A. (1981). Nisin: A possible alternative or adjunct to nitrite in the preservation of meats. Appl. Environ. Microbiol. 41, 375–380. 10.1128/aem.41.2.375-380.1981 7195188 PMC243702

[B118] Reis M. Eschbach-Bludau M. Iglesias-Wind M. I. Kupke T. Sahl H. G. (1994). Producer immunity towards the lantibiotic Pep5: Identification of the immunity gene pepI and localization and functional analysis of its gene product. Appl. Environ. Microbiol. 60, 2876–2883. 10.1128/aem.60.8.2876-2883.1994 8085827 PMC201737

[B119] Rhoads A. Au K. F. (2015). PacBio sequencing and its applications. Genomics Proteomics Bioinforma. 13, 278–289. 10.1016/j.gpb.2015.08.002 PMC4678779 26542840

[B120] Riley M. A. Wertz J. E. (2002). Bacteriocins: Evolution, ecology, and application. Annu. Rev. Microbiol. 56, 117–137. 10.1146/annurev.micro.56.012302.161024 12142491

[B121] Robichon D. Gouin E. Débarbouillé M. Cossart P. Cenatiempo Y. Héchard Y. (1997). The rpoN (sigma54) gene from Listeria monocytogenes is involved in resistance to mesentericin Y105, an antibacterial peptide from Leuconostoc mesenteroides. J. Bacteriol. 179, 7591–7594. 10.1128/jb.179.23.7591-7594.1997 9393729 PMC179715

[B122] Ross K. F. Ronson C. W. Tagg J. R. (1993). Isolation and characterization of the lantibiotic salivaricin A and its structural gene salA from Streptococcus salivarius 20P3. Appl. Environ. Microbiol. 59, 2014–2021. 10.1128/aem.59.7.2014-2021.1993 8357242 PMC182229

[B123] Ross R. P. Galvin M. McAuliffe O. Morgan S. M. Ryan M. P. Twomey D. P. (1999). Developing applications for lactococcal bacteriocins. Ant. Van Leeuwenhoek 76, 337–346. 10.1023/a:1002069416067 10532388

[B124] Schillinger U. Geisen R. Holzapfel W. H. (1996). Potential of antagonistic microorganisms and bacteriocins for the biological preservation of foods. Trends Food Sci. Technol. 7, 158–164. 10.1016/0924-2244(96)81256-8

[B125] Schillinger U. Kaya M. Lücke F. K. (1991). Behaviour of Listeria monocytogenes in meat and its control by a bacteriocin-producing strain of Lactobacillus sake. J. Appl. Bacteriol. 70, 473–478. 10.1111/j.1365-2672.1991.tb02743.x 1938671

[B126] Schoeman H. Vivier M. A. Du Toit M. Dicks L. M. Pretorius I. S. (1999). The development of bactericidal yeast strains by expressing the Pediococcus acidilactici pediocin gene (pedA) in *Saccharomyces cerevisiae* . Yeast 15, 647–656. 10.1002/(SICI)1097-0061(19990615)15:8<647::AID-YEA409>3.0 10392443

[B127] Severina E. Severin A. Tomasz A. (1998). Antibacterial efficacy of nisin against multidrug-resistant Gram-positive pathogens. J. Antimicrob. Chemother. 41, 341–347. 10.1093/jac/41.3.341 9578160

[B128] Siegers K. Entian K. D. (1995). Genes involved in immunity to the lantibiotic nisin produced by Lactococcus lactis 6F3. Appl. Environ. Microbiol. 61, 1082–1089. 10.1128/aem.61.3.1082-1089.1995 7793910 PMC167363

[B129] Siezen R. J. Rollema H. S. Kuipers O. P. de Vos W. M. (1995). Homology modelling of the Lactococcus lactis leader peptidase NisP and its interaction with the precursor of the lantibiotic nisin. Protein Eng. Des. Sel. 8, 117–125. 10.1093/protein/8.2.117 7630881

[B130] Sit C. S. Yoganathan S. Vederas J. C. (2011). Biosynthesis of aminovinyl-cysteine-containing peptides and its application in the production of potential drug candidates. Acc. Chem. Res. 44, 261–268. 10.1021/ar1001395 21366289

[B131] Stein T. Heinzmann S. Solovieva I. Entian K.-D. (2003). Function of Lactococcus lactis nisin immunity genes nisI and nisFEG after coordinated expression in the surrogate host Bacillus subtilis. J. Biol. Chem. 278, 89–94. 10.1074/jbc.M207237200 12379654

[B132] Szybalski W. Bryson V. (1952). Genetic studies on microbial cross resistance to toxic agents. I. Cross resistance of *Escherichia coli* to fifteen antibiotics. J. Bacteriol. 64, 489–499. 10.1128/jb.64.4.489-499.1952 12999676 PMC169383

[B133] Todorov S. D. Dicks L. M. T. (2004). Influence of growth conditions on the production of a bacteriocin by Lactococcus lactis subsp. lactis ST34BR, a strain isolated from barley beer. J. Basic Microbiol. 44, 305–316. 10.1002/jobm.200410413 15266603

[B134] Todorov S. D. Dicks L. M. T. (2005). Lactobacillus plantarum isolated from molasses produces bacteriocins active against Gram-negative bacteria. Enzyme Microb. Technol. 36, 318–326. 10.1016/j.enzmictec.2004.09.009

[B135] Valdés-Stauber N. Scherer S. (1994). Isolation and characterization of Linocin M18, a bacteriocin produced by Brevibacterium linens. Appl. Environ. Microbiol. 60, 3809–3814. 10.1128/aem.60.10.3809-3814.1994 7986050 PMC201890

[B136] Vaucher R. de A. Velho Gewehr C. de C. V. Correa A. P. F. Sant’Anna V. Ferreira J. Brandelli A. (2011). Evaluation of the immunogenicity and *in vivo* toxicity of the antimicrobial peptide P34. Int. J. Pharm. X. 421, 94–98. 10.1016/j.ijpharm.2011.09.020 21963470

[B137] Venema K. Haverkort R. E. Abee T. Haandrikman A. J. Leenhouts K. J. de Leij L. (1994). Mode of action of LciA, the lactococcin A immunity protein. Mol. Microbiol. 14, 521–532. 10.1111/j.1365-2958.1994.tb02186.x 7533883

[B138] Venema K. Venema G. Kok J. (1995). Lactococcal bacteriocins: Mode of action and immunity. Trends Microbiol. 3, 299–304. 10.1016/s0966-842x(00)88958-1 8528613

[B139] Venema K. Venema G. Kok J. (1995). Lactococcins: Mode of action, immunity and secretion. Int. Dairy J. 5, 815–832. 10.1016/0958-6946(95)00033-X

[B140] Vignolo G. Fadda S. de Kairuz M. N. de Ruiz Holgado A. A. Oliver G. (1996). Control of Listeria monocytogenes in ground beef by ‘Lactocin 705’, a bacteriocin produced by Lactobacillus casei CRL 705. a bacteriocin Prod. by Lact. casei CRL 705. Int. J. Food Microbiol. 29, 397–402. 10.1016/0168-1605(95)00038-0 8796440

[B141] Weyermann J. Lochmann D. Zimmer A. (2005). A practical note on the use of cytotoxicity assays. Int. J. Pharm. X. 288, 369–376. 10.1016/j.ijpharm.2004.09.018 15620877

[B142] Willey J. M. van der Donk W. A. (2007). Lantibiotics: Peptides of diverse structure and function. Annu. Rev. Microbiol. 61, 477–501. 10.1146/annurev.micro.61.080706.093501 17506681

[B143] Winkowski K. Crandall A. D. Montville T. J. (1993). Inhibition of Listeria monocytogenes by Lactobacillus bavaricus MN in beef systems at refrigeration temperatures. Appl. Environ. Microbiol. 59, 2552–2557. 10.1128/aem.59.8.2552-2557.1993 8368843 PMC182319

[B144] Yuan J. Zhang Z.-Z. Chen X.-Z. Yang W. Huan L.-D. (2004). Site-directed mutagenesis of the hinge region of nisinZ and properties of nisinZ mutants. Appl. Microbiol. Biotechnol. 64, 806–815. 10.1007/s00253-004-1599-1 15048591

[B145] Zhang T. Zhang Y. Li L. Jiang X. Chen Z. Zhao F. (2022). Biosynthesis and production of class II bacteriocins of food-associated lactic acid bacteria. Ferment. (Basel). 8, 217. 10.3390/fermentation8050217

[B146] Zottola E. A. Yezzi T. L. Ajao D. B. Roberts R. F. (1994). Utilization of cheddar cheese containing nisin as an antimicrobial agent in other foods. Int. J. Food Microbiol. 24, 227–238. 10.1016/0168-1605(94)90121-x 7703016

